# The circadian clock circuitry modulates leukemia initiating cell activity in T-cell acute lymphoblastic leukemia

**DOI:** 10.1186/s13046-023-02799-7

**Published:** 2023-08-24

**Authors:** Emanuele Murgo, Elisabetta De Santis, Francesca Sansico, Valentina Melocchi, Tommaso Colangelo, Costanzo Padovano, Mattia Colucci, Annalucia Carbone, Beatrice Totti, Alireza Basti, Lisa Gottschlich, Angela Relogio, Nazzareno Capitanio, Fabrizio Bianchi, Gianluigi Mazzoccoli, Vincenzo Giambra

**Affiliations:** 1 Department of Medical Sciences, Division of Internal Medicine and Chronobiology Laboratory, Fondazione IRCCS “Casa Sollievo Della Sofferenza”, San Giovanni Rotondo, FG 71013 Italy; 2 Hematopathology Unit, Fondazione IRCCS “Casa Sollievo Della Sofferenza”, San Giovanni Rotondo, FG 71013 Italy; 3 Cancer Biomarkers Unit, Fondazione IRCCS “Casa Sollievo Della Sofferenza”, San Giovanni Rotondo, FG 71013 Italy; 4 Institute for Systems Medicine, Faculty of Human Medicine, MSH Medical School Hamburg, Hamburg, 20457 Germany; 5 Present Address: Ivana Türbachova Laboratory for Epigenetics, Epiontis, Precision for Medicine GmbH, Berlin, Germany; 6 Molekulares Krebsforschungszentrum (MKFZ), Charité-Universitätsmedizin Berlin, Berlin, Germany; 7 Institute for Theoretical Biology (ITB), Charité-Universitätsmedizin Berlin and Humboldt-Universität Zu Berlin, Berlin, Germany; 8 Department of Clinical and Experimental Medicine, University of Foggia, Foggia, Italy

**Keywords:** T-ALL, Circadian, Biological clock, IL20R, STAT3, Flow cytometry

## Abstract

**Background:**

T-cell acute lymphoblastic leukemia (T-ALL) is an aggressive hematological malignancy, characterized by restricted cellular subsets with asymmetrically enriched leukemia initiating cell (LIC) activity. Nonetheless, it is still unclear which signaling programs promote LIC maintenance and progression.

**Methods:**

Here, we evaluated the role of the biological clock in the regulation of the molecular mechanisms and signaling pathways impacting the cellular dynamics in T-ALL through an integrated experimental approach including gene expression profiling of shRNA-modified T-ALL cell lines and Chromatin Immunoprecipitation Sequencing (ChIP-Seq) of leukemic cells. Patient-derived xenograft (PDXs) cell subsets were also genetically manipulated in order to assess the LIC activity modulated by the loss of biological clock in human T-ALL.

**Results:**

We report that the disruption of the circadian clock circuitry obtained through shRNA-mediated knockdown of *CLOCK* and *BMAL1* genes negatively impacted the growth in vitro as well as the activity in vivo of LIC derived from PDXs after transplantation into immunodeficient recipient mice. Additionally, gene expression data integrated with ChIP-Seq profiles of leukemic cells revealed that the circadian clock directly promotes the expression of genes, such as *IL20RB*, crucially involved in JAK/STAT signaling, making the T-ALL cells more responsive to Interleukin 20 (IL20).

**Conclusion:**

Taken together, our data support the concept that the biological clock drives the expression of IL20R prompting JAK/STAT signaling and promoting LIC activity in T-ALL and suggest that the selective targeting of circadian components could be therapeutically relevant for the treatment of T-ALL patients.

**Supplementary Information:**

The online version contains supplementary material available at 10.1186/s13046-023-02799-7.

## Background

T-cell acute lymphoblastic leukemia (T-ALL) is a variant of acute lymphoblastic leukemia (ALL) and accounts for 10%-15% of pediatric and up to 25% of adult ALL cases [1, 2]. Biologically T-ALL is hallmarked by genomic/genetic lesions impacting on a number of targetable pathways, comprising Notch, JAK/STAT, PI3K/Akt/mTOR, and MAPK signaling pathway [3, 4]. The main prognostic determinant in T-ALL is minimal residual disease (MRD) response and with current multi-chemotherapeutic schedules, outcomes for de novo T-ALL roughly span 80–85% 5-year event-free survival in the pediatric setting and overall survival is less than 50% in adult patients, but event-free and overall survival rates remain less than 25% for relapsed disease [2, 5]. This grim relapse-related prognosis critically advocates innovative therapeutic strategies for recurrent disease relapse prevention through therapy enhancing in high-risk patients, at the same time avoiding toxicity in subsets of patients with better prognosis [6].

Several critical signaling pathways involved in carcinogenesis including leukemogenesis are controlled by the biological clock, which drives circadian rhythmicity of crucial processes in normal and transformed cells going through malignant or potentially malignant changes [7–9]. The circadian clock circuitry plays an important role in the biology of lymphomas as well as chronic and acute myeloid or lymphocytic leukemia [10–14]. For instance, epigenetic changes of the *BMAL1* promoter were described in both B cell lymphoma and acute lymphocytic and myeloid leukemia [15], while PER2 was found downregulated in lymphoma and acute myeloid leukemia patients, with PER2 overexpression leading to cell cycle arrest and loss of clonogenicity [16]. Besides, PER2 up-regulation hampered human chronic myeloid leukemia cell proliferation in vitro and in vivo [17], while CRY1 upregulation was found in chronic lymphocytic leukemia [18], with its epigenetic silencing associated to an indolent clinical course [19].

The molecular clockwork is operated by protein-encoding genes whose expression fluctuates rhythmically with approximately 24-h (circadian) periodicity. These biological oscillators endow approximately every single cell of body tissues and organs and are organized in a hierarchical network structure coordinated by the master pacemakers located in the suprachiasmatic nuclei of the hypothalamus [20–22]. The circadian proteins hardwire a transcriptional/translational feed-back loop finely tuned by post-translational modifications, mainly kinase-ran phosphorylation [23]. Basically, the transcriptionally activating CLOCK:BMAL1 heterodimer sets off transcription of *PERIOD* (*PER1, 2, 3*) and *CRYPTOCHROME* (*CRY1, 2*) genes and their encoded proteins heterodimerize in the cytoplasm, pass back into the nucleus where hamper CLOCK:BMAL1 transcriptional activity. *BMAL1* expression is driven rhythmically by an auxiliary loop operated by the nuclear receptors REV-ERBα e RORα, which bind competitively at specific DNA sequences, termed ROR response elements (ROREs), on *BMAL1* promoter, with inhibitory and activating roles, respectively [23]. Another level of regulation of rhythmic gene expression is represented by reversible modifications in the chromatin structure, with reshaping dependent on epigenetic changes, such as histone post-translational modifications/replacements, including H3K27 acetylation–deacetylation and methylation–demethylation, which create a plastic framework of genetic transcription [23, 24].

A correctly functioning molecular clockwork has been shown to endow normal and malignant hematopoietic cells and the core transcription factors CLOCK and BMAL1 are essential for growth of acute myeloid leukemia cells [25]. Disruption of the molecular clockwork prompted differentiation with exhaustion of disease-propagating leukemia stem cells and genetic loss of *BMAL1* weakened acute myeloid leukemia preservation while normal hematopoiesis was not negatively impacted [25].

In this study, we aimed to explore the role of the circadian clock circuitry in T-ALL biology and investigate the prospective therapeutic benefits of targeting core cogs of the molecular clockwork. We report that the shRNA-mediated knockdown of *CLOCK* and *BMAL1* circadian genes negatively impacted the in vitro and in vivo cell expansion of human T-ALL cells. Additionally, gene expression data, integrated with Chromatin Immunoprecipitation Sequencing (ChIP-Seq) profiles of genetically modified leukemic cells, revealed that the circadian clock circuitry directly promoted the expression of genes, such IL20RB that encodes the β-subunit of Interleukin 20 (IL20) receptor, crucially involved in JAK/STAT signaling, making the T-ALL cells more responsive to IL20. Taken together, our data highlight the relevant role played by the core clock genes *CLOCK* and *BMAL1* alongside a proper functioning of the biological clock in the maintenance and progression of leukemia-initiating cells (LICs) in T-ALL.

## Methods

### Cell culture

Established human T-ALL cell lines (RPMI-8402 and SUP-T1) were cultured in RPMI 1640 medium supplemented with 10% fetal bovine serum (FBS), 1 mM sodium pyruvate, 2 mM L-glutamine, and antibiotics (Invitrogen). Xenograft-expanded primary human T-ALL cells were cultured on MS5-DL1 feeders in IMDM (GIBCO) with 10 ng/ml IL-2, 10 ng/ml IL-7 (Peprotech), and 0.75 uM SR1 (StemCell Technologies) as previously described [26]. Cell synchronization was performed by culturing T-ALL cell lines in RPMI 1640 medium supplemented with 50% FBS, 1 mM sodium pyruvate, 2 mM L-glutamine and antibiotics (Invitrogen) for two hours to express the protein simultaneously with an oscillatory pattern without altering the total protein level. Afterward cells were transferred in the completed RPMI 1640 medium supplemented with 10% FBS after washing in 1 × phosphate-buffered saline (PBS) [27]. The small molecule SR9011, which is a chemical agonist of the nuclear hormone receptors Rev-Erbα and Rev-Erbβ [28, 29], was resuspended in DMSO to a stock concentration of 10 mM, and then diluted serially in Hank’s Balanced Salt Solution (HBSS) before addition to culture media.

### Western blot

Whole cell lysates were generated after washing in ice-cold phosphate-buffered saline and then lysis in ice-cold 50 mM Tris–HCl (pH 7.4), 1% Nonidet P-40, 0.25% sodium deoxycholate, 150 mM sodium chloride, 1 mM sodium orthovanadate, 1 mM sodium fluoride, 2.5 mM sodium pyrophosphate, 1 mM EDTA, 1 mM phenylmethylsulphonyl fluoride, and protease inhibitor cocktail (cat #539,134, Calbiochem). Whole cell lysates or immunoprecipitated complexes were incubated at 95 °C for 10 min, loaded on SDS-PAGE gels and then transferred to Hybond-ECL membranes (Amersham). The membranes were blocked with 5% milk/0.3% TBS-Tween20 at 4 °C for 1 h and then probed with primary antibodies against BMAL1 (1:1,000 dilution; Cat. sc-48790, Santa Cruz Biotechnology), CLOCK (1:1,000 dilution; Cat. #5157, Cell Signaling Technology), CRY1 (1:1,000 dilution; Cat. sc-393466, Santa Cruz Biotechnology), CRY2 (1:1,000 dilution; Cat. sc-293263, Santa Cruz Biotechnology), PER1 (1:1,000 dilution; Cat. sc-25362, Santa Cruz Biotechnology), PER2 (1:1,000 dilution; Cat. sc-25363, Santa Cruz Biotechnology), RORα (1:1,000 dilution; Cat. sc-28612, Santa Cruz Biotechnology), Rev-Erbα (1:1,000 dilution; Cat. #13,418, Cell Signaling Technology), GAPDH (1:1,000 dilution; Cat. sc-365062, Santa Cruz Biotechnology) or β-Actin (1:6,000 dilution; Cat. #A1978, Sigma). HRP-conjugated secondary antibodies (Cat. NEF812001EA, Perkin Elmer) were used at 1:10 000 dilution. The chemiluminescent signal was detected with enhanced chemiluminescence (ECL) (cat. 32,106, Pierce) and subsequently with autoradiography. Band intensity was quantified using the ImageJ software [30].

### Co-Immunoprecipitation

Cells were lysed with NETN lysis buffer (Tris/HCl pH 7.5 50 mM, NaCl 150 mM, Np40 10ul, EDTA pH 8 0.5 mM, Proteinase Inhibitor (PMSF) 1 mM, Sodium Orthovanadate 100uM). The lysate was spun for 15 min, 14,000 rpm at 4 °C and subsequently incubated overnight at 4 °C with 125 ng of indicated antibody against BMAL1 (Cat. sc-48790, Santa Cruz Biotechnology), CLOCK (Cat. #5157, Cell Signaling Technology) or normal rabbit IgG (Cat. #2729, Cell Signaling) as a non-specific IgG control together with Dynabeads protein G (Cat. #10003D, ThermoFisher), previously blocked with 0.5 mg/ml BSA overnight at 4 °C. The immunocomplexes were then washed with NETN buffer and separated by SDS-PAGE.

### Plasmid and viral transduction

The RNAi Consortium (TRC) shRNAs targeting BMAL1 (shBMAL1_14, TRCN0000331014; shBMAL1_95, TRCN0000019095) or CLOCK (shCLOCK_76, TRCN0000018976; shCLOCK_77, TRCN0000018977) were cloned into a derivative of pLKO.1 vector (Addgene #8453) with the GFP or mCherry fluorescent marker. A cDNA encoding the human CLOCK, derived from Addgene construct (cod. #82,247) [31], was subcloned into a pRRL-based lentivector with truncated human NGFR (tNGFR) selection marker (PGK/tNGFR). All constructs were verified by sequencing. Lentiviruses were produced by transient co-transfection of 293 T cells with pCMVΔR8.74 and pCMV-VSV-G packaging/envelope vectors as described [32]. Lentivirus was concentrated by ultracentrifugation. Viral transduction was performed by spinfection in the presence of polybrene as described [33]. Virally transduced cells were sorted by flow cytometry as applicable.

### Transduction with lentiviral vectors for bioluminescence assay

For lentiviral transduction, 5 × 10^5^ cells were seeded into each well of a 24-well plate in 200µL. On the day of transduction, 300µL of supernatant of the corresponding lentivirus containing 8 μg/mL protamine sulfate (Sigma-Aldrich, St. Louis, MO, USA) and 4μg/mL polybrene (Sigma-Aldrich, St. Louis, MO, USA) was added to each well and mixed with the cell suspension. Cells were centrifuged at 300xg for 2 h at 33 °C. After spinoculation, cells were incubated in a humidified incubator at 37 °C with 5% CO2 for 2 days. After 2 days, cells were washed twice with fresh media and selection media (complete growth medium containing appropriate antibiotic) containing puromycin (1.5 µg/mL) was added to obtain stably transduced cells. Untransduced cells treated with the same antibiotic concentration were used as selection controls. Selection was performed until control cells died.

### Bioluminescence measurements

For live-cell bioluminescence recordings, 2.5 × 10^5^ cells were seeded in 35 mm dishes and maintained in phenol red-free RMPI (Gibco, Thermo Fisher Scientific, Waltham, MA, USA) containing 10% FBS, 1% Penicillin–Streptomycin, 1 mM sodium pyruvate, 2 mM GlutaMAX and supplemented with 250 µM D-Luciferin (Bio-Rad laboratories, Hercules, CA, USA). Cells were synchronized by FBS shock (50% FBS) for 2 h. *BMAL1*-promoter-reporter activities were measured using a LumiCycle instrument (Actimetrics, Wilmette, IL, USA) for five consecutive days. Raw luminescence data were de-trended by the 24 h running average (divided values) using the Chronostar analysis software V3.0 (Maier et al., 2022). The first 12 h of measurement were removed from the analysis, since the first data collection is comparatively very noisy due to technical limitations of the device.

### Flow cytometry assays

Human cells were stained with fluorochrome or biotin-conjugated antibodies against CD45 (eBioscience, ThermoFisher), anti-hCD271 (eBioscience, ThermoFisher) to detect the lentiviral tNGFR marker and DAPI (cat. D9542, Calbiochem, Sigma) for the exclusion of dead cells. We performed intracellular staining with an anti-phospho-STAT3 (Tyr705) APC-conjugated antibody (1:50 dilution; cat #17–9033-42, ThermoFisher) and an anti-STAT3 (pan) PE-conjugated antibody (1:50 dilution; cat #MA5-23,569, ThermoFisher) after paraformaldehyde fixation and permeabilization with 90% ice-cold methanol as specified by the manufacturer. We measured cell proliferation by BrdU incorporation according to the manufacturer’s instructions (BrdU kit, BD Biosciences). Absolute cell counts were determined using AccuCheck Counting Beads (cat #PCB100, ThermoFisher) following the manufacturer’s instructions. Early apoptotic cells were determined by AnnexinV binding and 7AAD exclusion using the PE Annexin V Apoptosis Detection Kit (Cat. No. 559763, BD PharmigenTM) and following the manufacturer’s recommendations. We performed FACS analysis and sorting on FACS Canto2, and MoFlo Astrios cell sorter (Beckman Coulter). We analyzed flow cytometry data using FlowJo software (Becton Dickinson). GraphPad-Prism 8.4.3 software was also employed for the visualization and statistical analyses of data.

### In vivo transplantation assays

Patient-derived xenografts were established by injection of primary patient biopsy material into irradiated NOD-Scid/IL2Rγc^−/−^ (NSG) mice as previously described [26]. The M71 and H3255 PDX lines were all reported previously [34]. The animals were housed in specific pathogen-free facilities at the Plaisant s.r.l. (Rome, Italy). The animal experiments were performed after protocol approval by the Institutional Review Boards of the Italian Ministry of Health, according to the Italian Regulation for Animal Health and Animal Welfare. Immunodeficient (NOD.Cg-Prkdcscid Il2rgtm1Wjl/SzJ, or NSG; RRID: IMSR_JAX:005557) xenograft recipient mice were 7–17 weeks of age. Male and female animals were represented in balanced proportion when in-house colony stock availability necessitated using mixed sex recipients.

### Gene expression analysis

Total RNA was isolated by TRIzol™ (Invitrogen) following manufacturer's standard protocol (TRIzol™ Reagent; Invitrogen). Gene expression profiling was performed using the Affymetrix GeneChip® Human Clarion S Array (Thermo Fisher Scientific) including more than 210,000 distinct probes representative of > 20,000 well-annotated genes. RNA samples have been amplified, fragmented, and labeled for array hybridization according to the manufacturer’s instruction. Samples were then hybridized ON, washed, stained, and scanned using the Affymetrix GeneChip Hybridization Oven 640, Fluidic Station 450 and Scanner 3000 7G (Thermo Fisher Scientific) to generate the raw data files (.CEL files). Quality control and normalization of Affymetrix.CEL files were performed using TAC software (v4.0; Thermo Fisher Scientific) by performing “Gene level SST-RMA” summarization method with human genome version hg38 (NCBI-Genome Reference Consortium Human Build 38). Gene expression data were Log2 transformed before analyses. Differentially expressed genes were defined as those with a fold-change (FC) difference of at least 1.5 and a *p*-value ≤ 0.05. Vulcano plots were generated by TAC software (v4.0; Thermo Fisher Scientific). Gene Set Enrichment Analysis was performed by using the “H:hallmark gene sets” collection (h.all.v2022.1.Hs.symbols.gmt; *N* = 50) or the “C7: immunologic signature gene sets (c7.immunesigdb.v2022.1.Hs.symbols.gmt; *N* = 4872), Diff_of_Classes as metric, with 1000 random gene sets permutation; the “median of probes” was used to collapse Affymetrix datasets to unique gene expression data. Bubble plot analysis of GSEA output was performed using JMP software (SAS Institute Inc., Cary, NC, USA). Ingenuity pathway analysis (IPA; QIAGEN, Hilden, Germany) was used to analyze and visualize canonical pathways enriched in BMAL1/Clock regulated genes. The Affymetrix analyses were performed in experimental replicates. Data were deposited in GEO database (https://www.ncbi.nlm.nih.gov/geo) with accession #: GSE225005.

### ChIP-sequencing

Chromatin immunoprecipitation (ChIP) was performed as described previously [35] with validated antibodies against BMAL1 (Cat. sc-48790, Santa Cruz Biotechnology), H3K27me3 (Cat. C15410069-50, Diagenode) and H3K27Ac (Cat. Ab4729, Abcam). ChIP-Seq libraries were prepared according to Accel-NGS 2S Plus DNA Library preparation kit (Cat. 21,024, Swift Biosciences). ChIP DNA was quantified using a Qubit dsDNA HS Assay Kit (Cat. Q32851, ThermoFisher Scientific) and the quality was assessed by the 4200 TapeStation System using High Sensitivity D1000 Reagents (Cat. 5067–5585, Agilent Technologies). DNA sequencing was performed on NextSeq500 Illumina System using a Mid Output Kit v2.5—150 cycles flow cell (Cat. 20,024,904, Illumina) for reaching about 10 million reads/sample. The ChIP-Seq data were aligned against human genome hg38 using STAR v2.7.0f with default parameters and filtered for removing reads aligning to ENCODE blacklist regions using sub-commands from the BedTools suit, the bedtools intersect. Peaks were called with Model-based Analysis for ChIP-seq (MACS2) [36] version 2.1.2 using default parameters after normalization with input as control. The peaks with *p*-value < 0.05 were retained. The ChIP-seq data were visualized using the Integrative Genomics Viewer (IGV). The peak overlaps between different ChIP-seq datasets were determined with the BedTools suit bedtools intersect. Peak annotation and genomic features (transcription starting sites TSS and genes associated with peaks) were found using the R Bioconductor package ChIPseeker [37]. The BAMM tool [38] was used to find shared consensus motives, 500 bp centered on each peak position. The ChIP-Seq data are accessible at NCBI SRA PRJNA932755.

### Statistics

GraphPad PRISM® 8.4.3 software was employed for the analyses of quantitative data, including the two-way ANOVA with Dunnett's test and the Fisher’s exact test, corrected for multiple comparisons by false discovery rate (FDR) using a two-stage linear step-up procedure of Benjamini, Krieger and Yekutieli [39]. LIC frequencies were calculated from limiting dilution transplant results as described [40].

## Results

### shRNA-mediated inhibition of CLOCK and BMAL1 gene expression compromises T-ALL cell growth

**Fig. 1:**
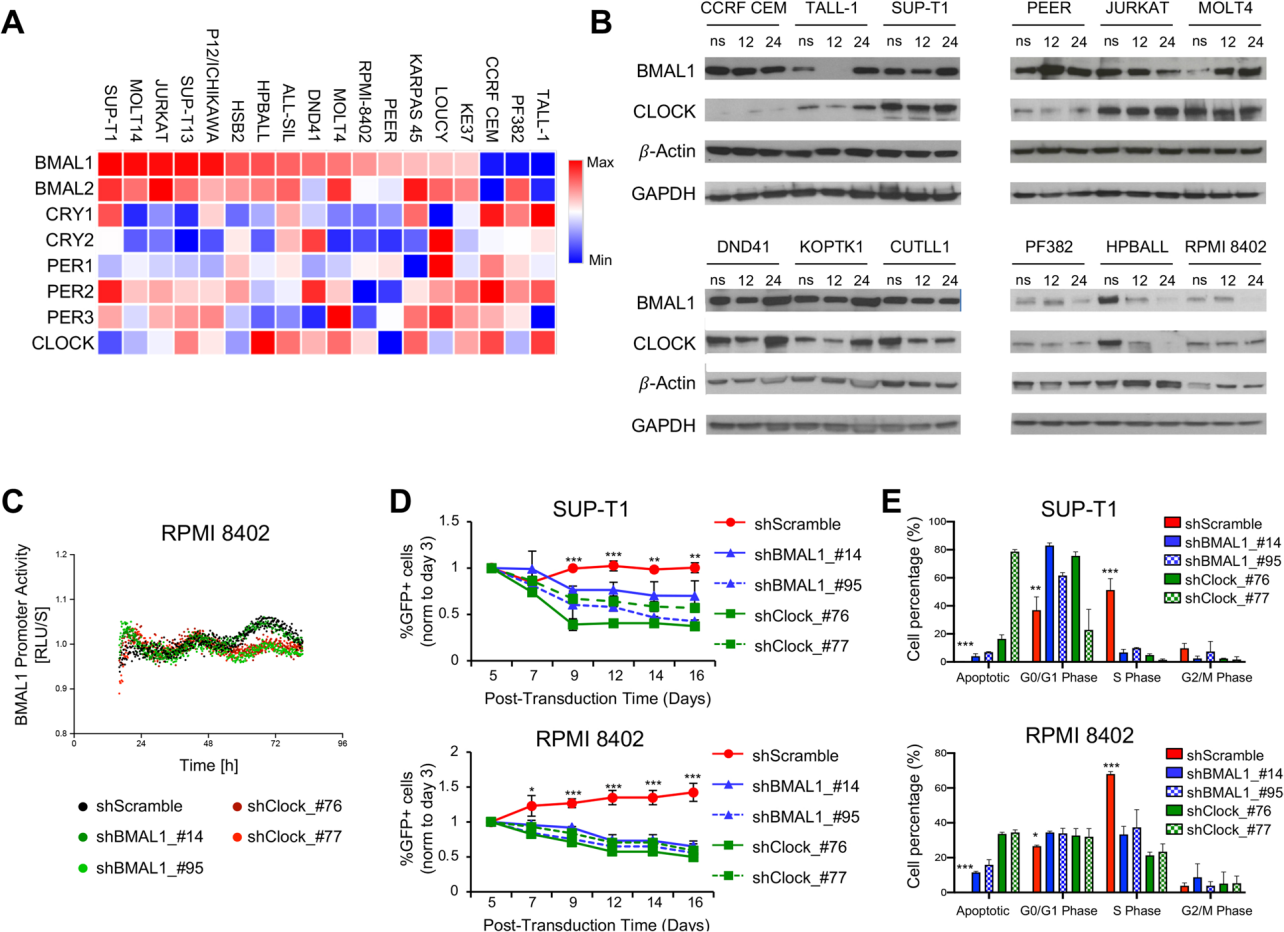
shRNA-mediated reduction of BMAL1 and CLOCK gene expression inhibits growth and cell proliferation in human T-ALL cell lines. **A** mRNA expression heatmap of eight core circadian clock genes in 18 T-ALL cell lines. Rlog values were calculated from RNA-seq data using DESeq2. Heatmap is scaled by gene (= row) with mean = 0 and SD = 1. Data are reanalyzed from the European Genome-phenome Archive (EGA) database under the accession code EGAS00001000536. **B** Western blot analysis of total BMAL1 and CLOCK in 12 human T-ALL cell lines. Whole cell lysates were generated from not synchronized (ns) cells and at 12 and 24 h after cell synchronization by culturing them in RPMI 1640 medium supplemented with 50% fetal bovine serum (FBS), sodium pyruvate (1 mM), L-Glutamine (2 mM) and antibiotics for two hours and then transferring them in the completed cell medium supplemented with 10% FBS. **C** Bioluminescence recordings of *BMAL1* promoter activity in synchronized RPMI 8402 control and shRNA modified cell lines. **D** Abundance of shRNA-transduced GFP + cell fraction, tracked over time in culture by flow cytometry. SUP-T1 and RPMI-8402 cell lines were independently transduced with two different clones of shRNA/GFP lentiviral constructs against BMAL1 or CLOCK genes or scramble control as indicated, FACS sorted and in vitro cultured. GFP + alive cells were measured at the indicated time points by flow cytometry for DAPI exclusion. The graphs report the result of three independent experiments performed in triplicate. *, p < 0.05; **, p < 0.01; ***, p < *0.001 (Two-way ANOVA with Dunnett's test, comparing the sh-scramble control mean with the other values)*. **E** Proliferation of SUP-T1 and RPMI-8402 cell lines following transduction with shBMAL1 or shCLOCK constructs or scrambled shRNA control as measured after 7 days of in vitro growth by BrdU incorporation. Apoptotic cells and populations in G1/G0, S and G2/M phases are gated as shown. The results depicted are representative of two independent experiments. *, p < 0.05; **, p < 0.01; ***, p < *0.001 (Two-way ANOVA with Dunnett's test, comparing the sh-scramble control mean with the other values)*

To address a potential role for the clock circadian circuitry in T-ALL biology, primarily we assessed the transcriptional expression of 8 core clock genes in human T-ALL cell lines using a publicly available dataset [41]. Analysis of RNA-Seq data revealed that the considered clock genes were expressed in all T-ALL cell lines examined, albeit at different levels (Fig. 1A). We chose to modulate the circadian genes CLOCK and BMAL1 in consideration of their fundamental role for the functioning of the biological clock. Therefore, through Western blot analysis, we evaluated the expression of total BMAL1 and CLOCK proteins in human T-ALL cell lines at 12 and 24 h after cell synchronization (Fig. 1B). We noted that BMAL1 and CLOCK protein levels varied among T-ALL cell lines and that levels of both transcription factors were higher in SUP-T1, JURKAT, MOLT4, DND41, KOPTK1 and CUTLL1 cell lines and lower in RPMI-8402, PF382 and HPBALL cells. Furthermore, we carried out co-immunoprecipitation experiments (co-IP) between BMAL1 and CLOCK protein to validate the physical interaction between both transcription factors (Fig. S1) and to corroborate the evidence that likewise non transformed cells, in T-ALL cells these two transcription factors regulate transcription as a heterodimer, as would be expected for cells with an intact circadian mechanism [42]. In RPMI-8402 and SUP-T1 cell lines, we also confirmed the protein expression of Period (Per1, Per2), Cryptochrome (Cry1, Cry2), Rev-Erbα nuclear receptor and RAR-related orphan receptor α (RORα), which are target genes of BMAL1:CLOCK heterodimer as well as core components of the molecular clockwork (Fig. S2). Subsequently, to test the need for a properly functioning biological clock in T-ALL cell lines as well as to assess whether leukemia T-cells are dependent on BMAL1 and CLOCK gene expression, SUP-T1 and RPMI-8402 cell lines were transduced with scramble control or shBMAL1 or shCLOCK constructs for knocking-down *BMAL1* and *CLOCK* gene, respectively (Fig. S3). In addition, for bioluminescence recordings we used a circadian reporter with a luciferase gene under control of BMAL1 promoter. Our bioluminescence recordings of *BMAL1* promoter activity in synchronized RPMI 8402 and SUP-T1 control and shRNA modified cell lines showed that downregulation of *BMAL1* and *CLOCK* leads to altered bioluminescence tracks, suggesting disruption of the biological clock (Fig. 1C and S4). Indeed, these results validate our hypothesis that a properly functioning biological clock endows leukemia T-ALL cell lines, which vitally depend on the activating elements of the molecular clockwork.

The circadian clock circuitry regulates the growth of normal and leukemia stem cells [25, 43]. To assess whether *BMAL1* and *CLOCK* are also required for cell growth and proliferation in T-ALL cells, shRNA-transduced SUP-T1 and RPMI-8402 cell lines were tracked over time in culture by flow cytometry. Interestingly, the shRNA-mediated decrease of BMAL1 and CLOCK gene expression in both T-ALL cell lines inhibited the in vitro expansion when compared to control cells (Fig. 1D). Moreover, to address whether *BMAL1* and *CLOCK* were able to promote cell proliferation, we assessed cell proliferation by Bromodeoxyuridine (BrdU) incorporation in shRNA-transduced SUP-T1 and RPMI-8402 cells and observed significant decrease of cycling activity, as well as increase of apoptotic cells over 7 days in culture following transduction with *BMAL1* and *CLOCK* shRNAs (Fig. 1E and S5-6). Taken together these results suggest that BMAL1 and its heterodimeric partner CLOCK are essential to upkeep cell proliferation and viability in human T-ALL cells.

### The circadian clock machinery regulates leukemia-initiating activity in T-ALL

**Fig. 2:**
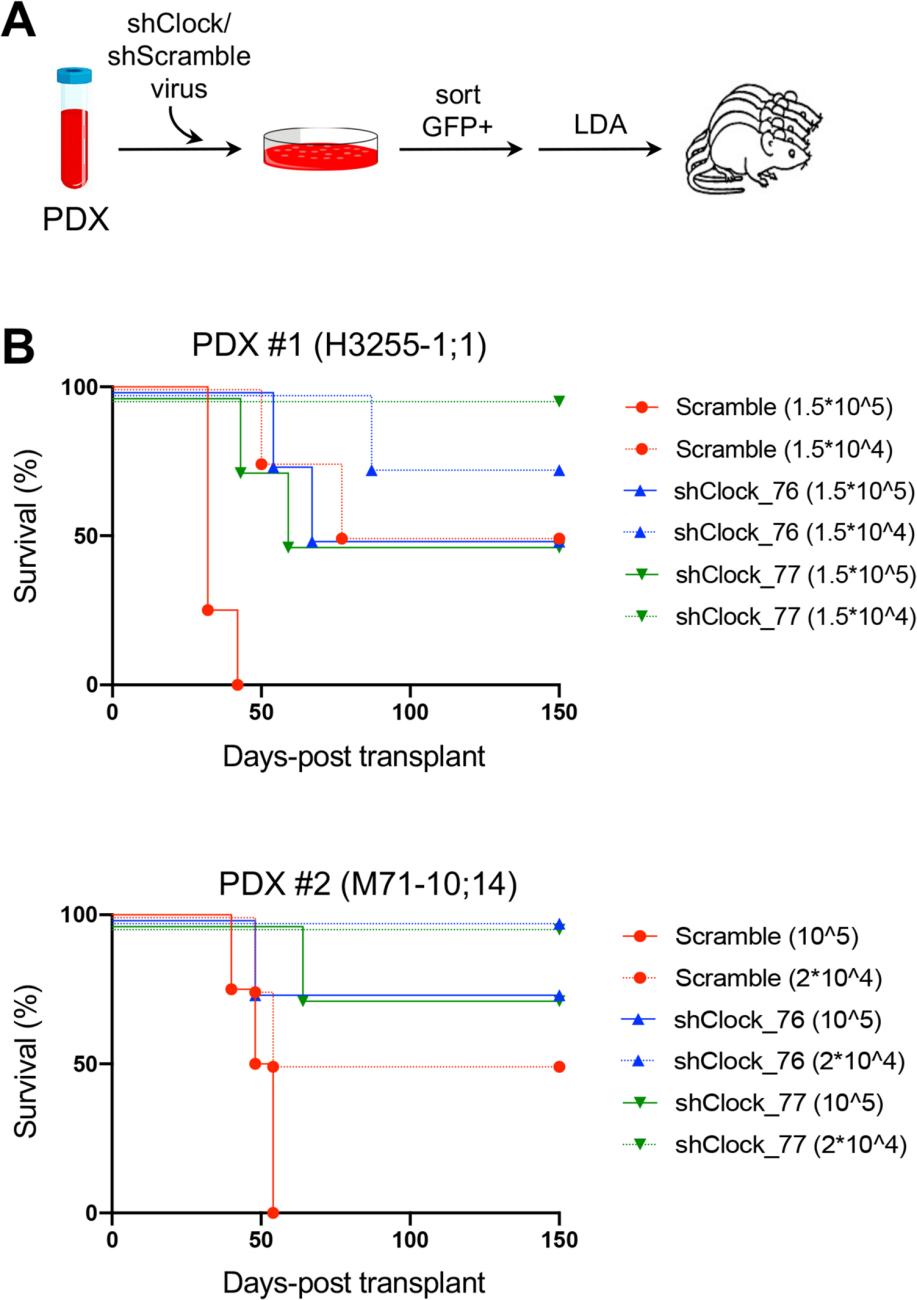
Patient-derived xenografts (PDXs) of T-ALL with reduced CLOCK expression have lower leukemia-initiating cell (LIC) frequency. **A** Schematic of transplantation experiments for limiting dilution analysis (LDA). PDX, Patient-derived xenograft. **B** Survival of recipient immunocompromised (NSG) mice after transplantation with FACS-sorted shCLOCK-transduced (GFP +) subsets from xenograft-expanded human leukemias. The cell doses injected in each of 4 recipient animals are indicated in parentheses. Two separate experiments are depicted using two independent PDX clones as indicated. The numbers of animals in each cohort and the raw data are available in Table S1

Our above-mentioned results suggest that BMAL1 and CLOCK promote cell-cycle progression and survival in T-ALL cells, consistent with findings reported in other cell systems [25, 44, 45]. To further explore the functional role of the circadian clock machinery with reference to the leukemia-initiating cell (LIC) propensity of established human T-ALLs, we selected two patient-derived xenografts (PDX) with high transcriptional and protein levels of BMAL1 and CLOCK transcription factors (Fig. S7) and introduced the shCLOCK constructs followed by GFP fluorescent marker through lentiviral transduction. After FACS-sorting purification, transduced cells were injected into secondary immunocompromised (NSG) recipient mice at limiting dilution. Strikingly, LIC activity was significantly decreased in the GFP + fraction of shCLOCK-transduced leukemia cells (Fig. 2 and Table S1). Specifically, the calculated LIC frequency in GFP + shScramble-transduced cells was 1 in 21,326 (95% CI: 1 in 5,547–82,258) and 1 in 24,967 (95% CI: 1 in 8,462–73,669) in PDX#1 and PDX#2 respectively; in GFP + shCLOCK_76-transduced cells was 1 in 180,624 (95% CI: 1 in 55,615–586,621) and 1 in 428,056 (95% CI: 1 in 61,011–3,003,261) in PDX#1 and PDX#2 respectively; and in GFP + shCLOCK_77-transduced cells was 1 in 216,404 (95% CI: 1 in 52,632–889,770) and 1 in 428,056 (95% CI: 1 in 61,011–3,003,261) in PDX#1 and PDX#2 respectively, as evaluated by ELDA analysis (see methods). Taken together, these data support the supposition that loss of CLOCK expression decreases LIC frequency in PDX of T-ALL cells as well as that a properly functioning circadian clock machinery promotes LIC activity in established human T-ALLs.

### Interleukin 20 receptor, β-subunit (IL20RB) is a direct target gene of the circadian clock machinery in T-ALL

**Fig. 3:**
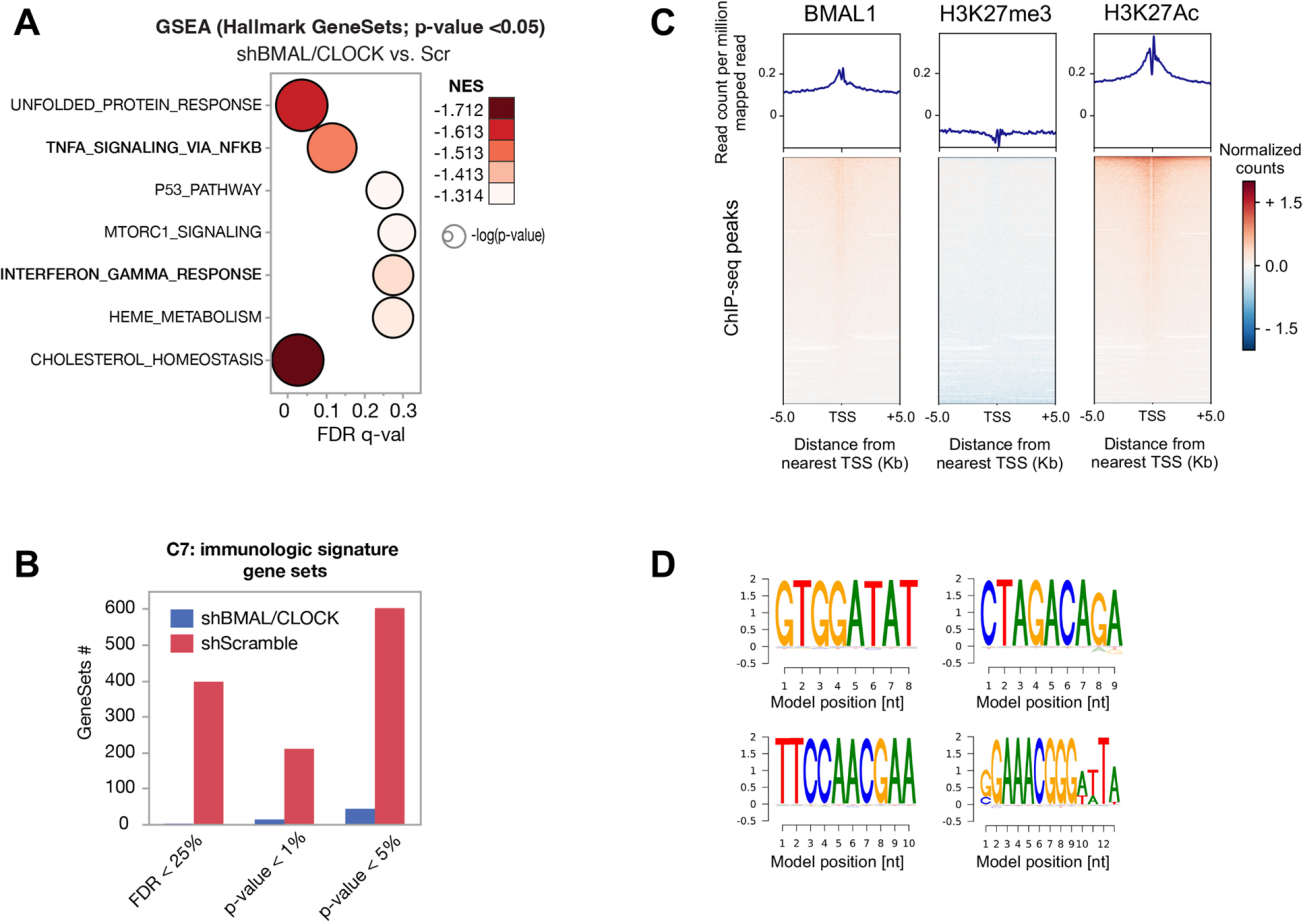
CLOCK and BMAL1 transcription factors promote the expression of genes involved in inflammatory pathways in T-ALL. **A** Bubble plot of the Hallmark GeneSets found negatively enriched in shBMAL1/CLOCK condition by Gene Set Enrichment Analysis (GSEA). Colors of the bubbles represent the magnitude of enrichment (NES, normalized enrichment score) and are as per the legend. Size of the bubbles represents the statistical significance of the enrichment (FDR, false-discovery-rate based on 1000 random permutation), *i.e.,* the bigger the most significant and are as per the legend. Y-axes, Gene Sets. X-axes, FDR of enrichment significance. **B** Bar plots represent the number of immunological Gene Sets (C7) enriched in T-ALL cells in the different conditions as per the legend. X-axes, indicate the different statistical significance of the enriched Gene Sets. **C** Average plot (top) and heatmap (bottom) of ChIP-Seq reads for BMAL1, H3K27me3 and H3K27Ac around the transcriptional starting site (TSS) in RPMI-8402 cell line. Upper panels show the average profile ± 5 kb around the centered TSS. Lower panels show read density heatmaps around the detected peak centers. Scales indicate normalized counts in the ChIP-Seq signal. **D** Position Weighted Matrix (PWM) logo identified by the BAMM suite platform (https://bammmotif.soedinglab.org/seed_results/423b6b96-fc2c-4ae3-a248-c2ea4a8eed7c/). The DNA motifs in the analyzed ChIP-seq data include the most frequent sites for BMAL1 in RPMI-8402 cell line

**Fig. 4:**
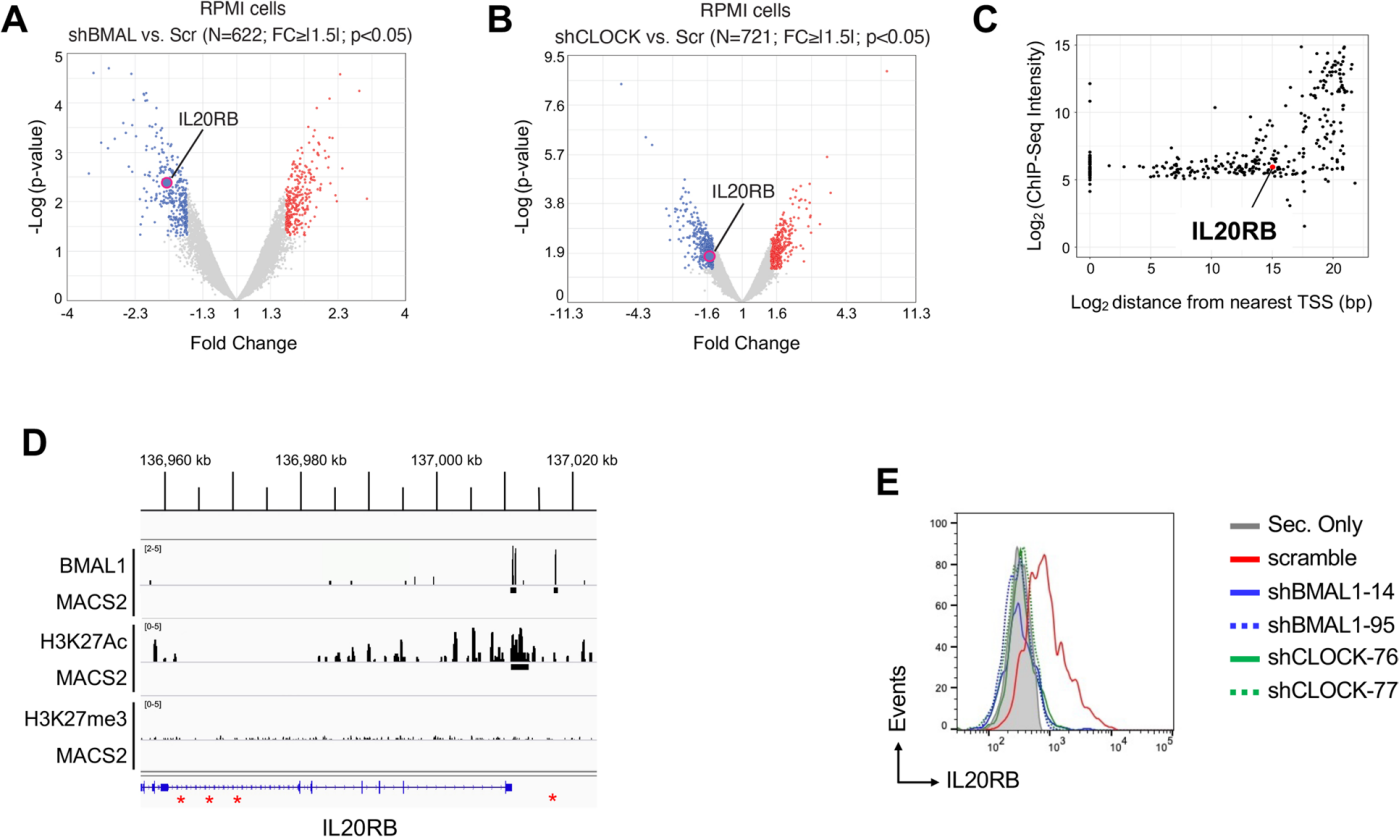
Interleukin 20 receptor, beta subunit (IL20RB) is a direct target gene of the circadian clock machinery in T-ALL. **A**-**B** Volcano plot of differentially expressed genes in shBMAL1 or shCLOCK (D) vs. shScramble T-ALL cells. Red (up-)/Blue(down-regulated) dots represent statistically significant genes (q-value < 0.05). N, number of significantly differentially expressed genes. FC, fold change (cutoff, |1.5|). **C** Location of predicted BMAL1 sites relative to transcription start sites (TSS) and ChIP-seq signal intensity (ChIP-seq score in Log2). In the plot, each dot represents BMAL1 peaks within 1 Kb around the TSS. The ChIP-seq signal over IL20RB gene in the RPMI-8402 cells is highlighted in red. **D** ChIPseq tracks for BMAL1, H3K27me3 and H3K27Ac over the human IL20RB 5’ region in the RPMI-8402 cells. Peaks of aligned reads over the IL20RB locus are shown along with MACS2 peak calls (*p*-value ≤ 0.05). The active genomic regions are identified by the H3K27Ac histone mark. The canonical “E-box” binding site for CLOCK-BMAL1 in the IL20RB locus are highlighted in red. **E** Protein expression level of Interleukin 20 receptor (IL20R) by flow cytometric analysis in the SUP-T1 cells transduced with two different clones of shRNA/GFP lentiviral constructs against BMAL1 or CLOCK genes or scramble control as indicated and in vitro cultured for 7 days. GFP + alive cells were measured after seven days from the transduction and identified for DAPI exclusion by flow cytometry

To investigate the impact on human T-ALL transcriptome of the modulation of CLOCK and BMAL1, we performed microarray gene expression profiling of RPMI-8402 cells transduced with two different clones of shRNA/GFP lentiviral constructs against *BMAL1* or *CLOCK* genes or scramble control. Overall, a total of 622 genes (FC >|1.5|; *p* < 0.05; see methods) were found differentially regulated in shBMAL1 condition vs. control (Table S2) while 721 genes (FC >|1.5|; *p* < 0.05; see methods) were found differentially regulated in shCLOCK condition (Table S3). Then we investigated the biomolecular mechanisms mostly affected by such transcriptional rewiring by performing Gene Set Enrichment Analysis (GSEA) using Hallmark Gene Set (*N* = 50; see methods). A total of 7 gene sets were found significantly downregulated (NES < -1.3; *p* < 0.05), consequently suggesting the inactivation of the relative biomolecular functions in shBMAL1 and shCLOCK condition when compared to control cells (Fig. 3A). Interestingly, we found that TNF-α/NF-kB signalling and IFNγ response were among these 7 gene sets. This result prompted us to further investigate the role of the circadian clock machinery in the regulation of immune phenotypes in the context of T-ALL. GSEA performed using a large collection of immunole-associated gene signatures (C7; *N* > 4500) revealed a global downregulation of these signatures in shBMAL1 and shCLOCK condition, suggesting a global impairment of immune phenotype and function (Fig. 3B). To highlight in T-ALL cells direct binding sites and target genes of the main circadian transcriptional activator, we generated ChIP-seq data for BMAL1, H3K27me3 and H3K27Ac in the RPMI-8402 cell line. We found 332 DNA sequences associated to active genomic regions identified by the H3K27Ac histone mark around the transcriptional starting sites (TSS) (Fig. 3C). We identified consensus motifs for BMAL1-CLOCK transcription factors, including canonical DNA response elements called enhancer box (E-box) [46] as well as novel predicted binding sites (Fig. 3D). We focused on 5 top gene transcripts, highly down-regulated in the shBMAL1- and shCLOCK-transduced cells as compared to control cell conditions (Fig. 4A-B and Table S4) and enriched in DNA binding sites for BMAL1 (Fig. 4C). Of interest, we noted that a BMAL1 binding site was also present in the active genomic region over the TSS of *IL20RB* gene (Fig. 4D), whose expression was significantly decreased by shRNA-mediated knock-down of BMAL1 or CLOCK gene in RPMI-8402 cells (Fig. 4A-B). Furthermore, we corroborated the specific modulation of IL20R by BMAL1 and CLOCK transcription factors in SUP-T1 cell line, which showed higher level of IL20R in the cell surface than RPMI-8402 cells by flow cytometry analysis (Fig. 4E and S8), confirming that the circadian clock machinery promotes IL20R expression in human T-ALL cells.

### CLOCK promotes cell proliferation in T-ALL cells through IL20R signaling

**Fig. 5:**
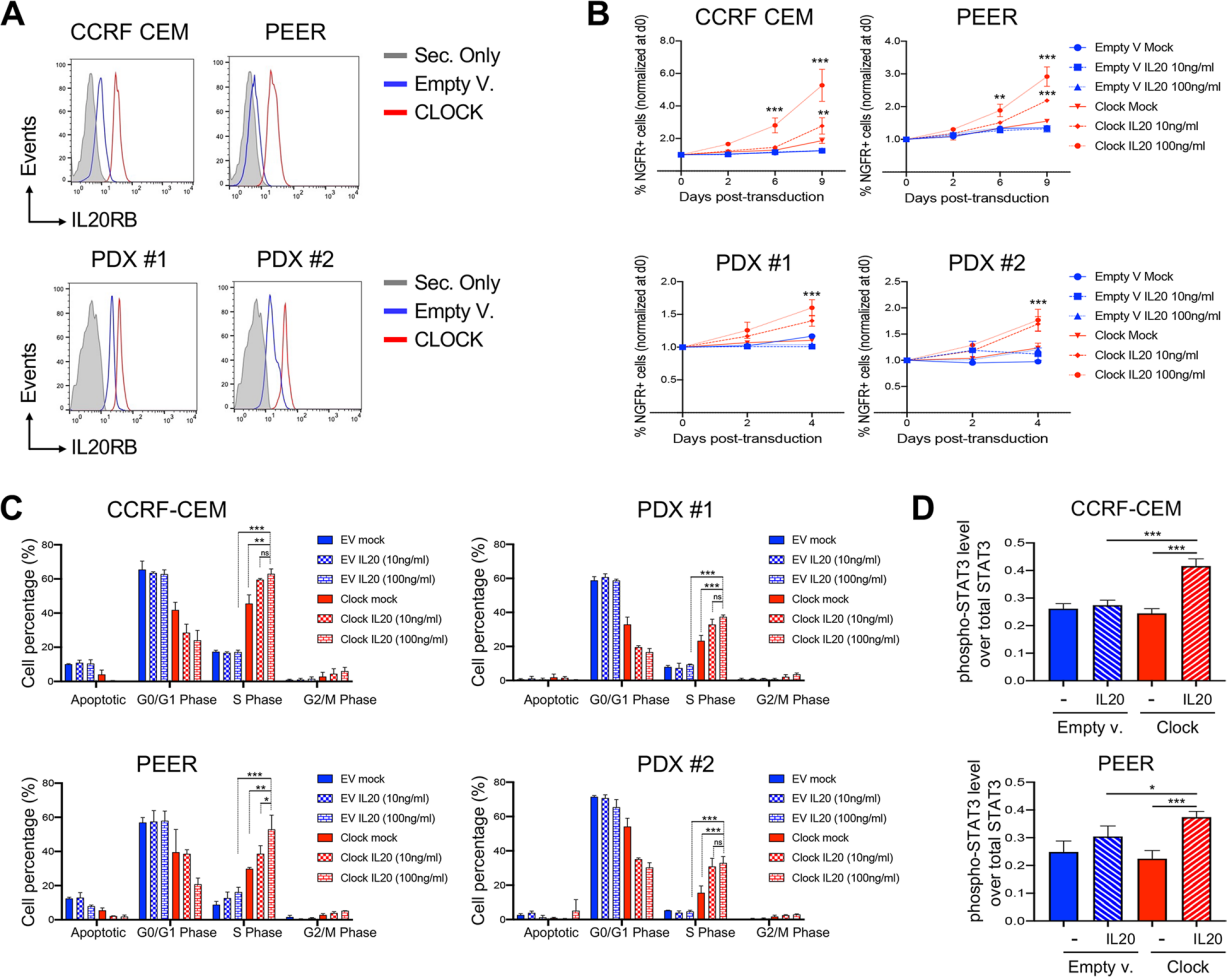
CLOCK promotes cell proliferation in T-ALL cells through IL20R signaling. **A** Protein expression level of IL20RB by flow cytometry in CCRF-CEM and PEER cell lines and PDX models transduced with CLOCK or empty (EV) lentivectors with truncated human NGFR (tNGFR) selection marker. NGFR + alive cells were measured after three days from the transduction and identified for DAPI exclusion by flow cytometry. **B** Flow cytometry analysis of abundance of NGFR + alive cell fraction after transduction with CLOCK or empty (EV) lentivectors as reported in (A) and following treatments with IL20 at the indicated concentration (10 ng/ml and 100 ng/ml) or phosphate-buffered saline (PBS) as mock control. Transduced subsets of CCRF-CEM and PEER cell lines and PDX models were tracked over time at the indicated time points by flow cytometry. Alive NGRF + cells were discriminated for DAPI exclusion. Means ± SD fraction of the initial transduction value are plotted for experiments performed in triplicate. **, p < 0.01; ***, p < *0.001 (Two-way ANOVA with Dunnett's test, comparing the mean of CLOCK-transduced IL20-treated cells, with the other values)*. **C** Flow cytometric analysis of cell proliferation by BrdU incorporation in human CCRF-CEM and PEER cell lines and PDXs, following transduction with CLOCK or empty lentivectors as indicated. Transduced cells were treated with IL20 or mock control as reported in (B) and measured after three days of in vitro IL20 stimulation by flow cytometry. The graphs report the result of two independent experiments performed in triplicate. ns, not significant; **, p < 0.05; **, p < 0.01; ***, p < *0.001 (Student's t-test)*. **D** Protein expression level of total and phosphorylated proteins of STAT3 in CCRF-CEM and PEER cell lines transduced with CLOCK or empty lentivectors and following 72 h incubation with or without IL20 as indicated Each graph reports the ratio of mean fluorescence intensity (MFI) of phosphorylated protein over the MFI of total protein in two independent experiments. *, *p* < 0.05; **, p < 0.01; ***, p < *0.001 (Student's t-test)*

To further confirm that in human T-ALL the circadian clock machinery promotes IL20R expression, CCRF-CEM and PEER cell lines and two independent clones of PDXs were transduced with lentiviruses carrying CLOCK gene, or empty vector as control, together with the truncated human Nerve growth factor receptor (tNGFR) as a selection marker. CLOCK overexpressing T-ALL cells were assessed by flow cytometry for evaluating expression of IL20R at protein level. In agreement with our previous findings, we observed that in both human T-ALL cell lines and PDX samples of human T-ALL, the constitutive expression of CLOCK significantly increased the expression of IL20R at protein level as compared to control cells (Fig. 5A). Furthermore, to investigate the functional axis of CLOCK and IL20R, transduced cell lines and PDX samples were cultured with either 10 ng/ml or 100 ng/ml of human-IL20 (hIL20) or in media lacking cytokine (mock) (Fig. 5B). Interestingly, we found that the addition of recombinant hIL20 to CLOCK-overexpressing T-ALLcells induced greater expansion with respect to mock treated cells, as well as control cells transduced with empty vector (Fig. 5B). In response to stimulation with IL20 in vitro, we also observed a pronounced cycling activity of CLOCK-transduced leukemia T-cells (Fig. 5B), as well as decrease of the fraction of cells at the early apoptotic stage (Fig. S9), confirming that CLOCK-overexpressing cells are more responsive to IL20 treatment.

The binding of IL20 ligand to IL20R activates a main pathway including the Janus kinase (JAK)/ signal transducers and activator of transcription (STAT), supporting cell survival and growth [47, 48]. Notably, our data resulting from gene expression profiling suggest that genes enriching JAK/STAT signaling pathway are directly driven by BMAL1 and CLOCK transcription factors (Fig. S10). To corroborate this hypothesis and to determine how IL20R promotes T-ALL cell expansion and cycling activity, we assessed in CLOCK-transduced T-ALL cells by flow cytometry the protein level of the downstream mediator p-STAT3. We discovered that, upon CLOCK overexpression, IL20R protein level increased and in response to stimulation by IL20 STAT3 phosphorylation and activation occurred (Fig. 5D). Taken together, these data support the concept that the CLOCK-IL20R-JAK/STAT functional axis might promote leukemia T-cell proliferation and prime T-ALL cell expansion and could represent a crucial pathogenic mechanism in the context of human T-ALL.

### The expression level of IL20RB and genes related to JAK/STAT signaling pathway are highly correlated with CLOCK transcriptional level in T-ALL patients

**Fig. 6:**
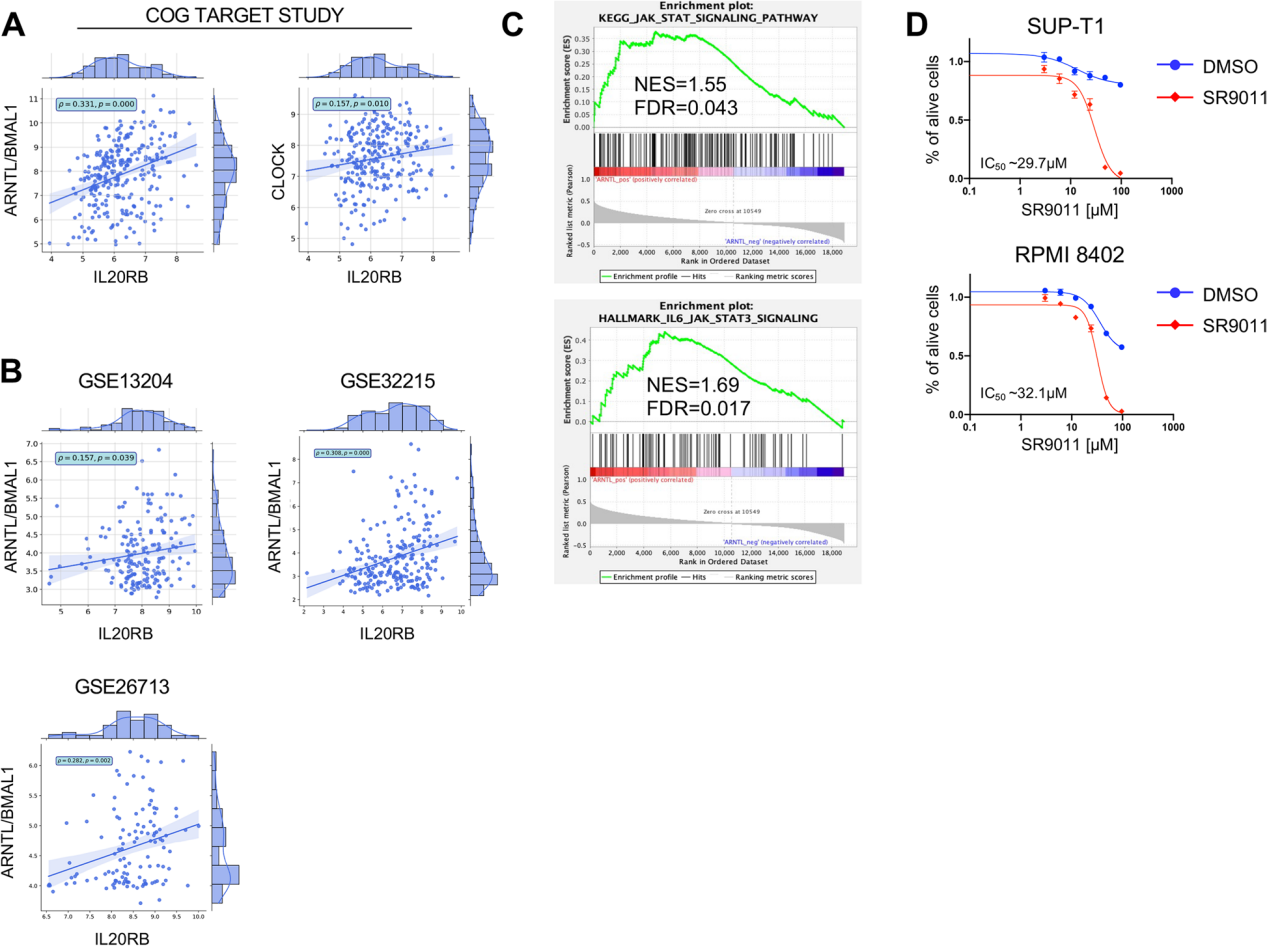
Primary patient T-ALL samples with high ARNTL mRNA level have high gene expression of L20RB transcript ad well as genes related to JAK/STAT signaling pathway. **A** Correlation analysis for ARNTL/BMAL1 vs. IL20RB and CLOCK vs. IL20RB mRNA expression level (normalized rLog) among 264 T-ALL patients from the COG TARGET study [4]. Pearson correlation coefficient (ρ) and *p*Value (p) are also reported in the plot. **B** Correlation analysis for ARNTL/BMAL1 and IL20RB expression level (normalized with RMA (Robust Multiarray Averaging) Affymetrix microarray (HG-U133 Plus 2.0) data was downloaded from Gene Expression Omnibus (accessions GEO: GSE13204 (*n* = 174), GSE32215 (*n* = 228) and GSE26713 (*n* = 117)). Probe sets for ARNTL/BMAL1 and IL20RB were identified using a custom CDF file. Pearson correlation coefficient (ρ) and *p*Value (p) are also reported in the plot. **C** Gene set enrichment analysis (GSEA). The gene signature was derived from the differential expression analysis of RNA-seq data sets from the COG TARGET study [4]. All genes were ranked for the differential expression of ARNTL/BMAL1 gene in high vs. low T-ALL patients. NES, normalized enrichment score; FDR, false discovery rate. **D** Dose–response curve showing relative viability (y axis) of human SUP-T1 and RPMI-8402 cell lines treated in vitro with variable concentrations of SR9011 agonist of the nuclear hormone receptors Rev-Erbα and Rev-Erbβ and inhibitor of BMAL1 transcription (x axis) (*n* = 3). Viable cells were measured after 72 h of treatment with DMSO (mock) or SR9011 at the indicated concentrations by flow cytometry analysis for DRAQ7 exclusion. The graphs report the result of two independent experiments performed in duplicates (mean ± SD are plotted)

To assess the relevance of the afore-mentioned results for T-ALL patients in the clinical setting, we evaluated the transcriptional level of CLOCK, BMAL1 and IL20RB genes in a cohort of 264 diagnostic T-ALL patients’ samples from the COG TARGET study [4]. Interestingly, we found a statistically significant correlation of IL20RB expression with both CLOCK and BMAL1 mRNA levels (Fig. 6A). We confirmed this statistically significant correlation between BMAL1/IL20RB and CLOCK/IL20R expression levels in three large T-ALL patients cohorts, totaling 519 patients’ samples [49–52] (Fig. 6B), supporting the relevance of this functional axis in T-ALL patients as well. To further characterize T-ALL patients with high *BMAL1* mRNA expression at transcriptional level, we performed gene expression enrichment analysis (GSEA) using two different gene set collections representing JAK/STAT signaling pathway. Interestingly, we found that T-ALL patients with high *BMAL1* gene levels showed significant positive enrichment for the genes of JAK/STAT signaling pathway (Fig. 6C), corroborating the significance of the interplay between circadian pathway and JAK/STAT signaling in T-ALL patients.

**Fig. 7:**
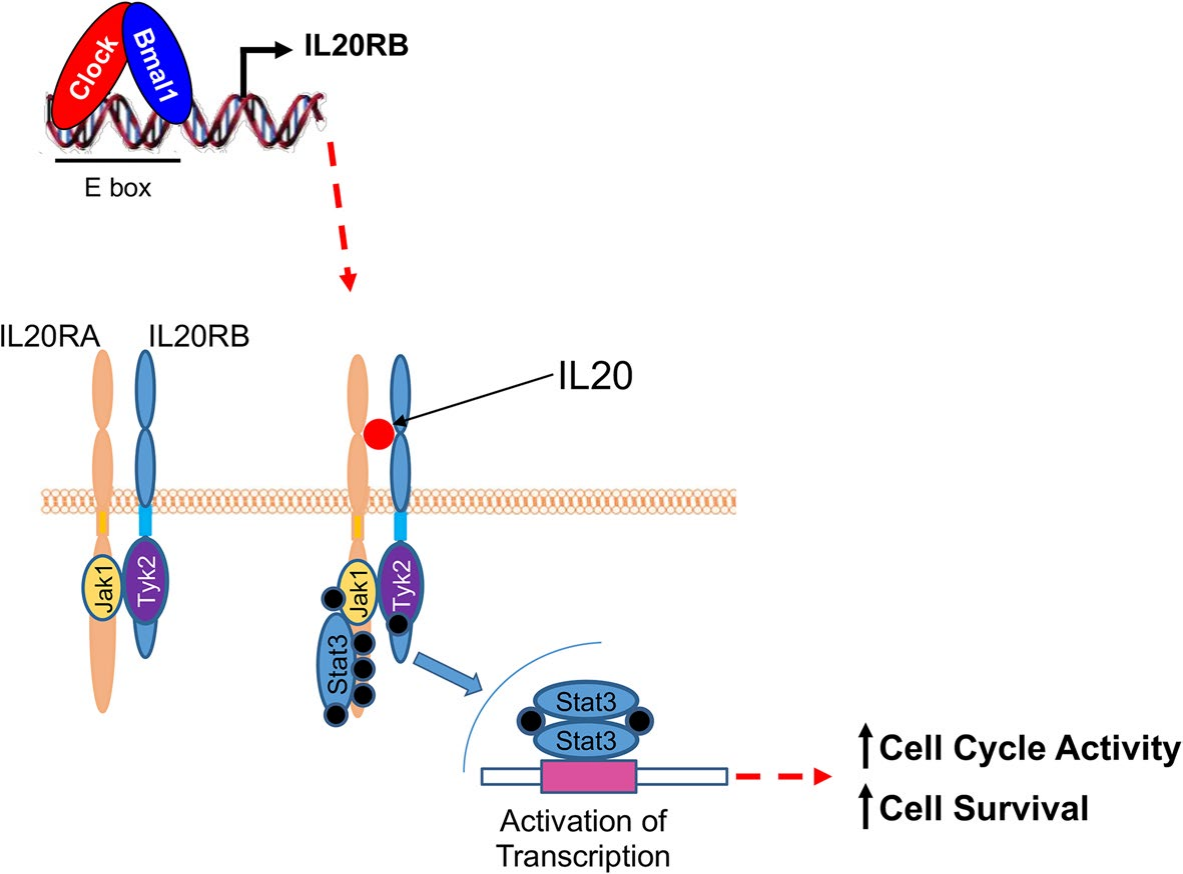
Schematic of the molecular mechanism involving the circadian clock machinery and the IL20R signaling pathways through the STAT mediators in the maintenance of leukemia initiating cell activity in T-ALL

Our data suggest that the disruption of the circadian clock circuitry in T-ALL might be therapeutically relevant. To address this hypothesis, SUP-T1 and RPMI-8402 human T-ALL cell lines were treated for three days with SR9011, a specific agonist of the nuclear hormone receptors Rev-Erbα and Rev-Erbβ,which inhibit BMAL1 transcription [28, 29]. In agreement with our above-mentioned observations, we found a dose-dependent decrease in cell viability for both cell lines (IC_50_ = 29.7 μM for SUP-T1 and IC_50_ = 32.1 μM for RPMI-8402) (Fig. 6D). In addition, we observed that constitutive expression of CLOCK gene in CCRF-CEM and PEER cell lines decreased the pro-apoptotic effect induced by SR9011, when compared to empty vector (EV) transduced control cells (Fig. S11). Taken together, these data support the concept that alteration of the circadian clock circuitry influences the maintenance and progression of T-ALL cells through decreased activation of IL20R and JAK/STAT signaling pathway (Fig. 7) and that disruption of the circadian clock circuitry might be therapeutically relevant.

## Discussion

T-ALL is an aggressive and rapidly progressive form of hematological malignancy characterized by the clonal proliferation of immature T-cell precursors, featured by defined cellular subsets with asymmetrically enriched LIC activity. At present, the signaling pathways upholding LIC maintenance and progression in this neoplasm of lymphoblasts committed to T-lineage are not completely known [53, 54]. Factually, prognosis of T-ALL patients is worse respect to other ALL patients, considering that T-ALL patients are at increased risk for induction failure, early relapse, and isolated central nervous system relapse and poor salvage, with less than 25% event-free and overall survival rates for relapsed disease [55]. This implies the need of deeply inquiring prognostic biomarkers and druggable molecular mechanisms to improve stratification and therapeutic strategies for T-ALL patients at high risk for treatment failure [56]. The biological clock plays a crucial role in controlling the proliferative and differentiating dynamics of immunocompetent and hematopoietic cells and endows normal and malignant cells. A properly ticking molecular clockwork is needed for acute myelogenous leukemia (AML) cell growth and its derangement induces leukemia stem cell differentiation and hinders AML maintenance with normal hematopoiesis preservation [25].

In our study, we evaluated the role of the biological clock in the regulation of the molecular mechanisms and signaling pathways impacting the cellular dynamics in T-ALL. We first evaluated the expression of core clock genes in the transcriptome of T-ALL cell lines and analyzed the expression of BMAL1 and CLOCK proteins in several of these cell lines in the presence and absence of molecular clockwork synchronization. Based on the expression of these circadian proteins we selected the RPMI and SupT1 cell lines, which showed different expression levels of both BMAL1 and CLOCK proteins. In these cell lines we then evaluated bioluminometric tracks, cell cycle and proliferative dynamics in control conditions and after silencing of BMAL1 and CLOCK genes. The results consistently indicated that the silencing of the main activators of the biological clock impacts the phenotype of the T-ALL cell lines, disturbing the proper ticking of the molecular clockwork. This is shown by modification of the oscillation profile as rendered by continuous real-time bioluminescence recording of a promoter-coupled luciferase reporter, and by significant decrease of proliferative capacity with blockade of cells in G0/G1 phase and increase in the cell fraction that undergoes apoptosis. The interference on the biological clock obtained by challenge with a chemical agonist of the REV-ERBα nuclear receptor confirmed the crucial role played by BMAL1 in favoring cell proliferation in T-ALL and the effective pharmacological effect of this chemical compound, as evaluated by measurement of the half maximal inhibitory concentration (IC_50_).

The important role of the alteration of the biological clock in inducing reduction of the proliferative capacity of T-ALL cells was then confirmed with experiments carried out in a murine model, which showed longer survival of immunodeficient recipient mice after transplantation of leukemia T-cells obtained from patient-derived xenografts (PDXs) silenced for BMAL1 or CLOCK. The bioinformatics analysis performed on the transcriptome of BMAL1 and CLOCK-silenced SupT1 and RPMI cells showed deactivation of cytokine-dependent signaling and in particular down-regulation of the gene coding for the β-subunit of IL20 receptor (IL20RB), as also confirmed by cytofluorimetric analysis. Furthermore, in our study we used different cell lines for the various experiments due to methodological topics. RPMI 8402 and SupT1 cells were used in CLOCK and BMAL1 downregulation experiments because they reliably express CLOCK and BMAL1 (SupT1 at high level, RPMI at medium level), then we used CCRF CEM and PEER cells that express CLOCK at low level to evaluate changes of cell phenotype upon CLOCK upregulation and IL20 challenge. In other experiments we used primary T-ALL cells (PDX1 and PDX2: patients derived xenograft cells) to evaluate more translational T-ALL cell models.

IL20 is a member of the IL10 subfamily (IL19, IL20, IL22, IL24 and IL26), featured by conjoint biology based on structural homology and shared receptor usage. IL-20 signals through class II cytokine receptors, a twelve proteins family featured by amino acid residues conservation patterns in the extracellular protein domains. In particular, the IL20 receptor is a heterodimeric receptor complex consisting of α and β subunits, encoded by IL20RA and IL20RB genes, respectively [57–59]. The β-subunit of its receptor (IL20RB) plays a key role in the activation of intracellular signaling involving JAK/STAT with STAT3 phosphorylation and activation of nuclear transcription of genes coding for factors involved in the control of cell proliferation and induction of aggressive neoplastic phenotype [57–59]. Interestingly, IL20RB gene was found expressed with circadian rhythmicity in mouse tissues [60] and previous reports showed that Cryptochrome proteins drive circadian rhythmicity of pro-inflammatory cytokines secretion [61]. Accordingly, in our study ChIP-Seq profiling performed with an antibody directed against BMAL1 confirmed binding to the promoter of the IL20RB gene, in correspondence also with the binding localization for H3K27Ac and H3K27me3 traits, indicating permissive/repressive epigenetic modifications at the level of the IL20RB promoter, respectively. Bioinformatics analysis of the T-ALL patients database [4] showed a statistically significant positive correlation of BMAL1 and IL20RB expression levels, corroborating the clinical relevance of this genetic/genomic and functional interplay. To confirm the role of the biological clock in determining the phenotype of T-ALL cells, we overexpressed CLOCK in T-ALL cell lines characterized by its reduced basal expression and in primary T-ALL lines. CLOCK gene over-expression induced increased expression of the β-subunit of the IL20 receptor and of the phosphorylated form of STAT3, indicating activation of the JAK/STAT signaling pathway. This result is important considering the relevant role played by JAK/STAT signaling, a targetable pathway, in supporting T-ALL cell proliferation and impacting disease outcome [6]. The phenotypic modification of CLOCK-overexpressing T-ALL cells was featured by increased proliferative capacity, with increased cell fraction in S and G2/M phase and decreased tendency to apoptosis in response to increasing doses of IL20. This result was further confirmed by decreased pro-apoptotic effect of SR9011, a pharmacological agonist of the nuclear receptor REV-ERBα and blocker of BMAL1, when evaluated by measurement of the half maximal inhibitory concentration (IC50).

## Conclusions

On the basis of previous evidence that, differently from healthy hematopoietic cells, the leukemia stem cells fully rely on a properly functioning molecular clockwork for survival and growth, we evaluated the role of the core circadian transcription factors BMAL1 and CLOCK in T-ALL biology. We showed that T-ALL cells harbor an intact circadian clock circuitry and the correct ticking of the biological clock is necessary to maintain T-ALL cells growth and self-renewal. This action takes place through IL20-dependent activation of JAK/STAT signaling with distinct involvement of the β-subunit of the IL20 receptor, whose expression is directly and rhythmically driven by BMAL1 binding on the IL20RB gene promoter. Our results support an original pro-tumorigenic role for the circadian clock circuitry in T-ALL biology and open the way to further studies focusing on the circadian transcription factors BMAL1 and CLOCK as useful biomarkers for prognostic stratification and the biological clock as potential target for innovative therapeutic strategies in T-ALL patients.

### Supplementary Information

**Additional file 1.**

**Additional file 2.**

**Additional file 3.**

## Data Availability

The mRNA expression data can be accessible at NCBI GEO (GSE225005; reviewer token: ydqdoyeihrshngt). Top gene transcripts (*N* = 622) differentially expressed upon shBMAL1 or shCLOCK in RPMI-8402 can be found in Table S2 and S3 respectively. The ChIP-Seq data are accessible at NCBI SRA PRJNA932755. Top gene transcripts, highly down-regulated in the shCLOCK/BMAL1-transduced cells as compared to control cell conditions and bound by BMAL1 in RPMI-8402 cell line are available in Table S4. For other original data, please contact the correspondent author.

## Abbreviations

T-ALL: T-cell acute lymphoblastic leukemia
LIC: Leukemia initiating cell
ChIP-Seq: Chromatin immunoprecipitation sequencing
PDX: Patient-derived xenografts
IL20: Interleukin 20
IL20RB: β-Subunit of IL20 receptor
MRD: Minimal residual disease
RORE: ROR response elements
PBS: Phosphate-buffered saline
FBS: Fetal bovine serum
PER: Period
CRY: Cryptochrome
BrdU: Bromodeoxyuridine
NSG: NOD.Cg-Prkdcscid Il2rgtm1Wjl/SzJ mice
GSEA: Gene set enrichment analysis
E-box: Enhancer box
tNGFR: Truncated nerve growth factor receptor
JAK: Janus kinase
STAT: Signal transducers and activator of transcription
EV: Empty vector
IC_50_: Half-maximal inhibitory concentration
EGA: European Genome-phenome Archive
SD: Standard deviation
LDA: Limiting dilution analysis
NES: Normalized enrichment scores
FDR: False-discovery-rate
TSS: Transcriptional starting site
PWM: Position weighted matrix
MFI: Mean fluorescence intensity
RMA: Robust multiarray averaging
GEO: Gene expression omnibus
