# Supplementary material for: The circadian clock circuitry modulates leukemia initiating cell activity in T-cell acute lymphoblastic leukemia
Source: J Exp Clin Cancer Res. 2023 Aug 24;42:218. doi: 10.1186/s13046-023-02799-7 (PMC10464343; doi:10.1186/s13046-023-02799-7)
Supplement: Supplementary file 1 — Additional file 1. [file 13046_2023_2799_MOESM1_ESM.pdf]

The circadian clock circuitry modulates leukemia initiating cell activity in T-cell acute lymphoblastic leukemia

Emanuele Murgo[1,*], Elisabetta De Santis[2,*], Francesca Sansico[2], Valentina Melocchi[3], Tommaso Colangelo[3], Costanzo Padovano[2], Mattia Colucci[2], Annalucia Carbone[1], Beatrice Totti[2], Angela Relogio[4,5], Nazzareno Capitanio[6], Fabrizio Bianchi[3], Gianluigi Mazzoccoli[1,*,±], Vincenzo Giambra[2,*,±]

[1]Department of Medical Sciences, Division of Internal Medicine and Chronobiology Laboratory, Fondazione IRCCS "Casa Sollievo della Sofferenza", 71013 San Giovanni Rotondo (FG), Italy

[2]Hematopathology Unit, Fondazione IRCCS "Casa Sollievo della Sofferenza", 71013 San Giovanni Rotondo (FG), Italy

[3]Cancer Biomarkers Unit, Fondazione IRCCS "Casa Sollievo della Sofferenza", 71013 San Giovanni Rotondo (FG), Italy

[4] Molekulares Krebsforschungszentrum (MKFZ), Charité-Universitätsmedizin Berlin, Germany

[5]Institute for Theoretical Biology (ITB), Charité-Universitätsmedizin Berlin and Humboldt-Universität zu Berlin, Berlin, Germany

[6]Department of Clinical and Experimental Medicine, University of Foggia, Foggia, Italy

Supplemental Information including:

Figures S1 to S11

Table S1 and Table Legends S2 to S4

Supplementary Figures

Fig. S1.

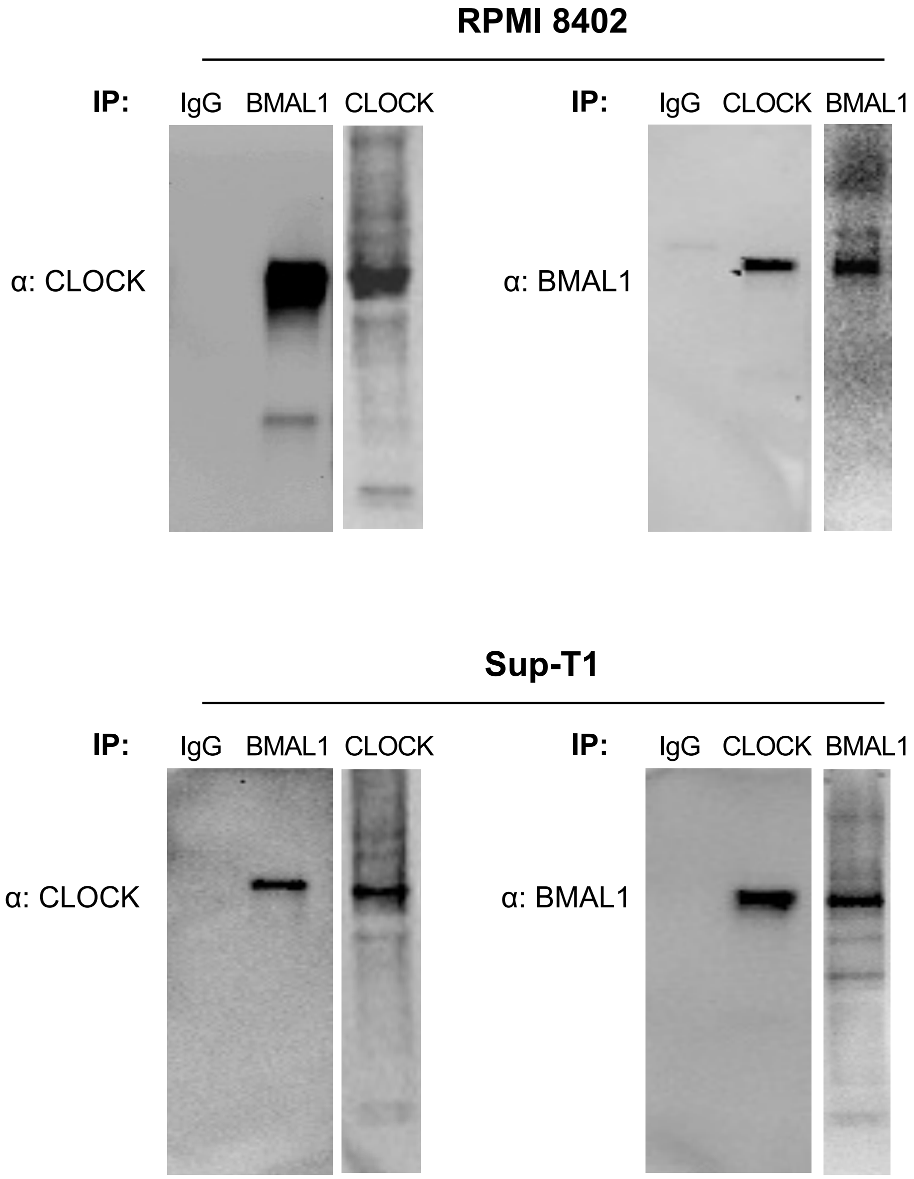

Figure S1. Co-Immunoprecipitation (Co-IP) between BMAL1 and CLOCL in RPMI-8402 and SUP-T1 cell lines, followed by immunoblot analysis using the indicated antibodies.

Fig. S2.

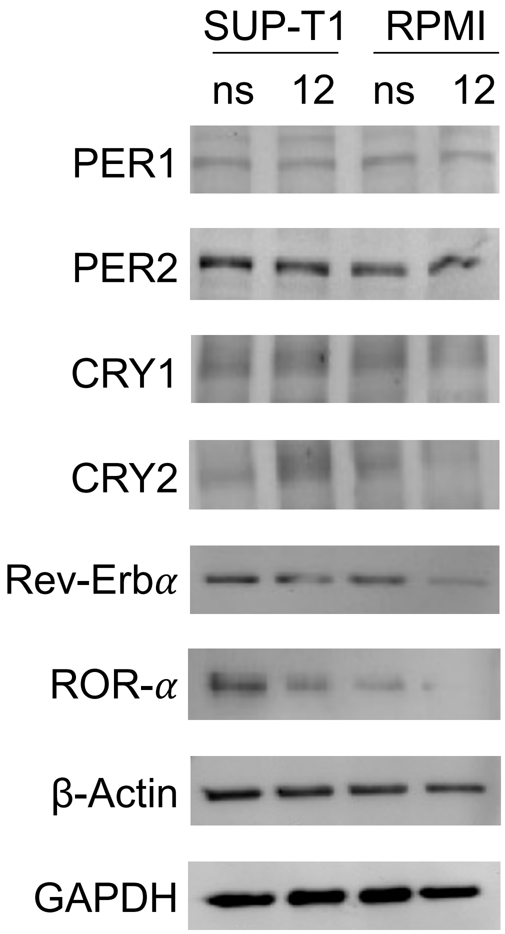

Figure S2. Western blot analysis of core circadian protein factors in SUP-T1 and RPMI-8402, human T-ALL cell lines.

Whole cell lysates were generated from not synchronized (ns) cells and at 12 hours after cell synchronization by culturing the human cells in RPMI 1640 medium supplemented with 50% fetal bovine serum (FBS), sodium pyruvate (1 mM), L-Glutamine (2 mM) and antibiotics for two hours and then transferring them in the completed cell medium supplemented with 10% FBS.

Fig. S3.

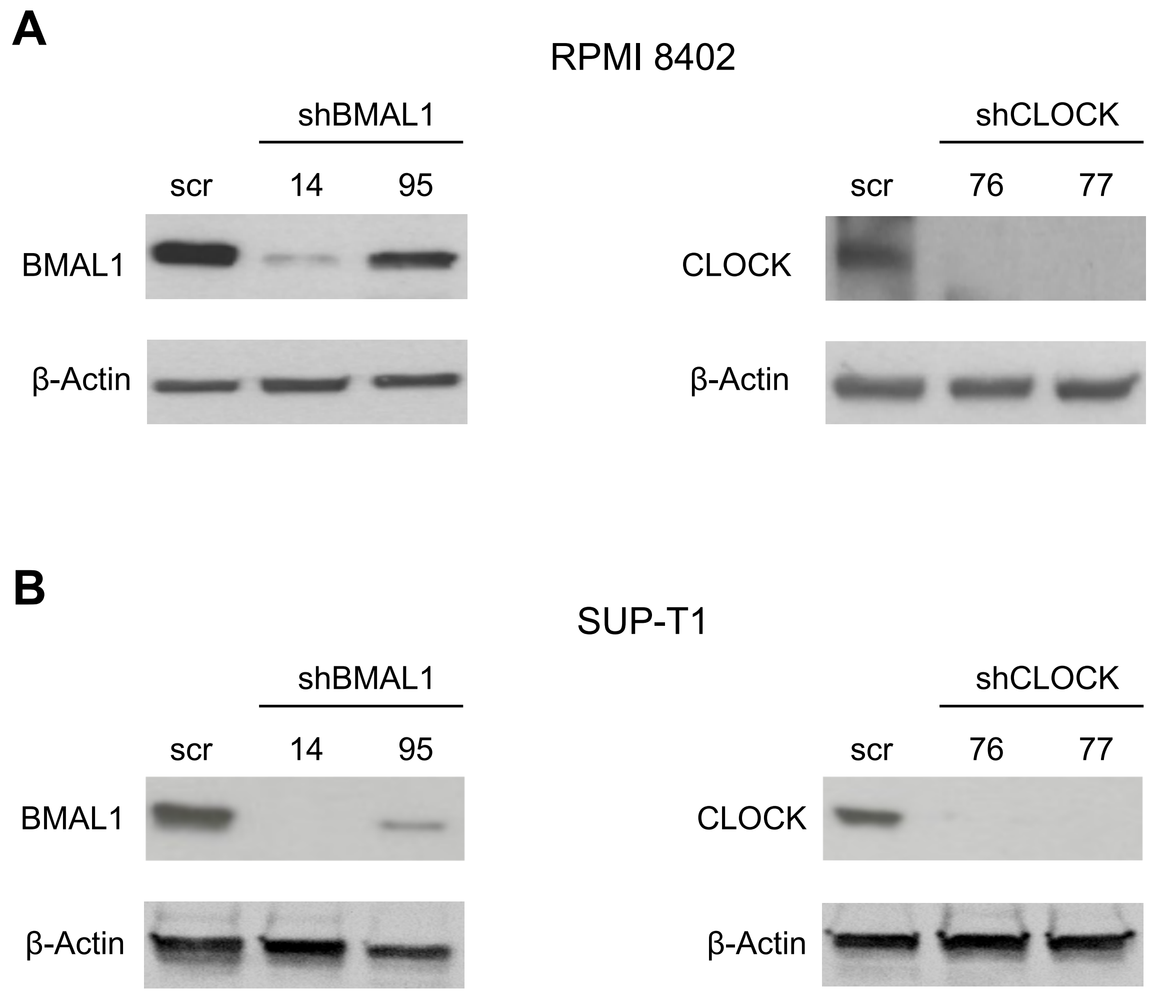

Figure S3. Expression level of BMAL1 and CLOCK proteins in RPMI-8402 and SUP-T1 cells, transduced with shScramble, shBMAL1 or shCLOCK lentivectors.

(**A-B**) Western blot analyses of total BMAL1 and CLOCK protein levels in the RPMI-8402 (**A**) and SUP-T1 (**B**) human T-ALL cell lines following knock-down of BMAL1 or CLOCK by transduction with lentiviral shRNAs or scrambled negative control (shScr).

Fig. S4.

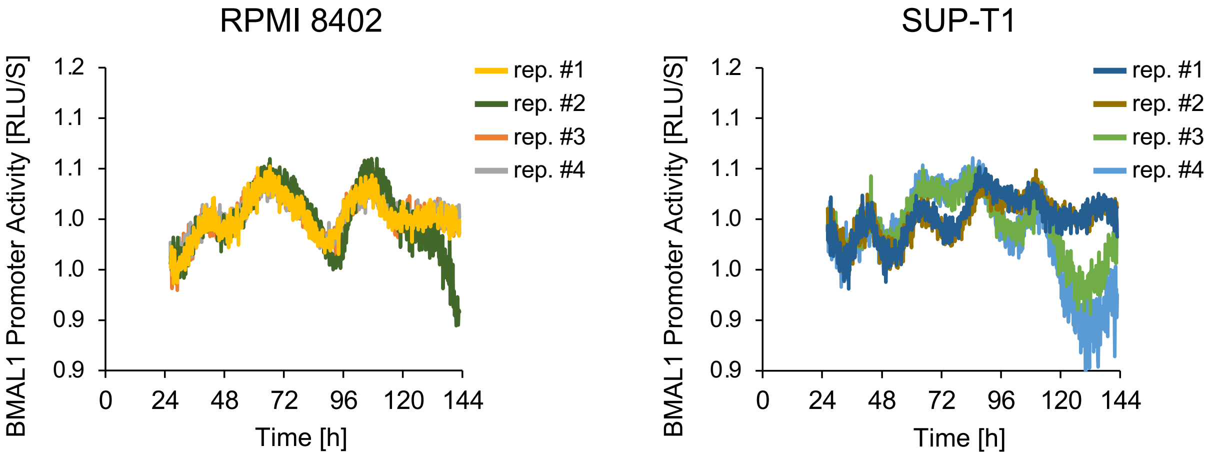

Figure S4. Bioluminescence recordings of BMAL1 promoter activity in synchronized RPMI 8402 and SupT1 cell lines. In each plot, biological replicates for each cell line are depicted in different colours (n=4).

Fig. S5.

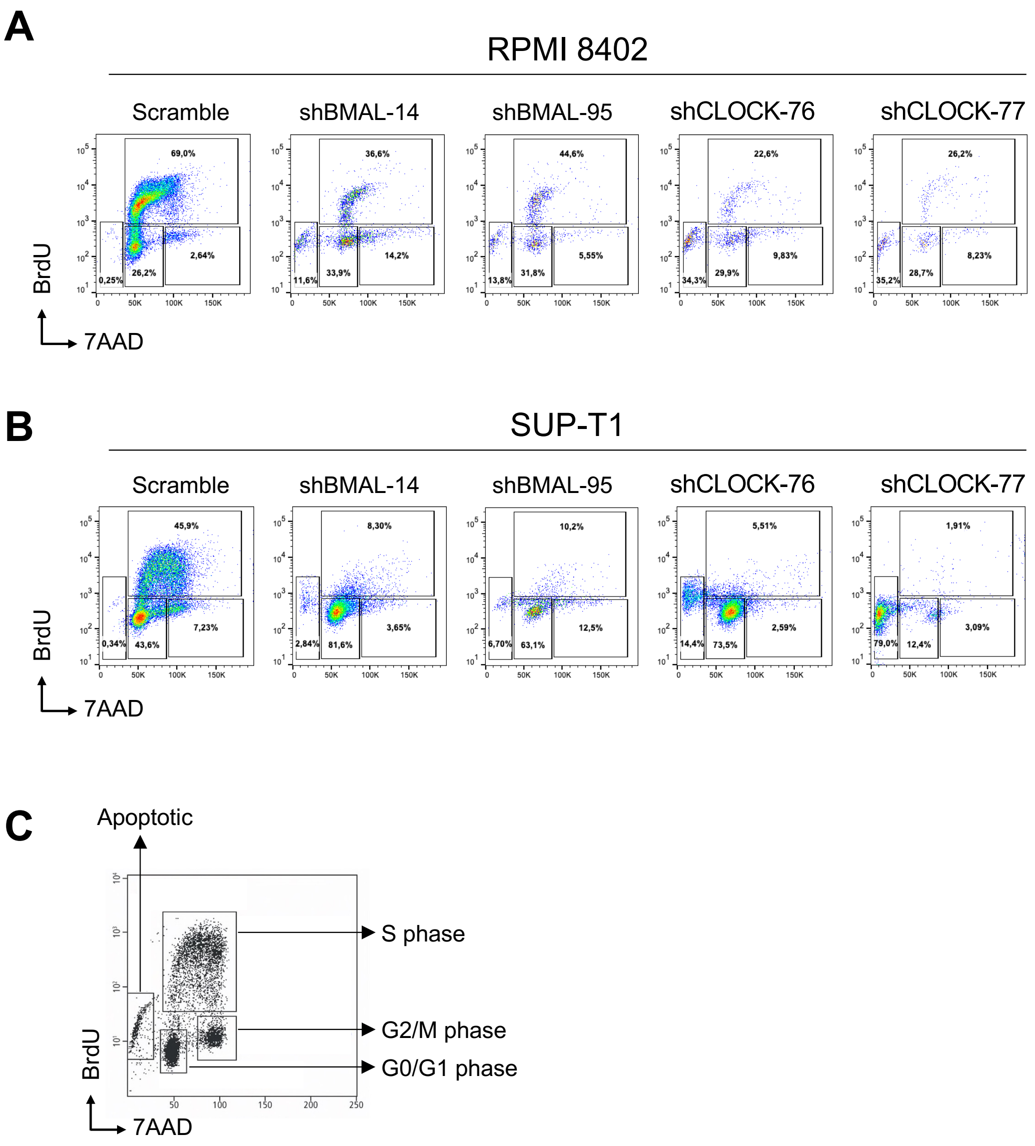

Figure S5. Flow cytometry plots of BrdU assay used to generate the bar charts reported in Figure 1E for RPMI-8402 and SUP-T1 human T-ALL cell lines.

(**A-B**) BrdU incorporation of RPMI-8402 (**A**) and SUP-T1 (**B**) cell lines was assessed after 7 days of in vitro growth following transduction with shBMAL1 or shCLOCK constructs or scrambled shRNA control as measured by flow cytometry. (**C**) Gating strategy for the identification of cell subsets in cell cycle and apoptosis as determined by BrdU incorporation and total DNA levels.

Fig. S6.

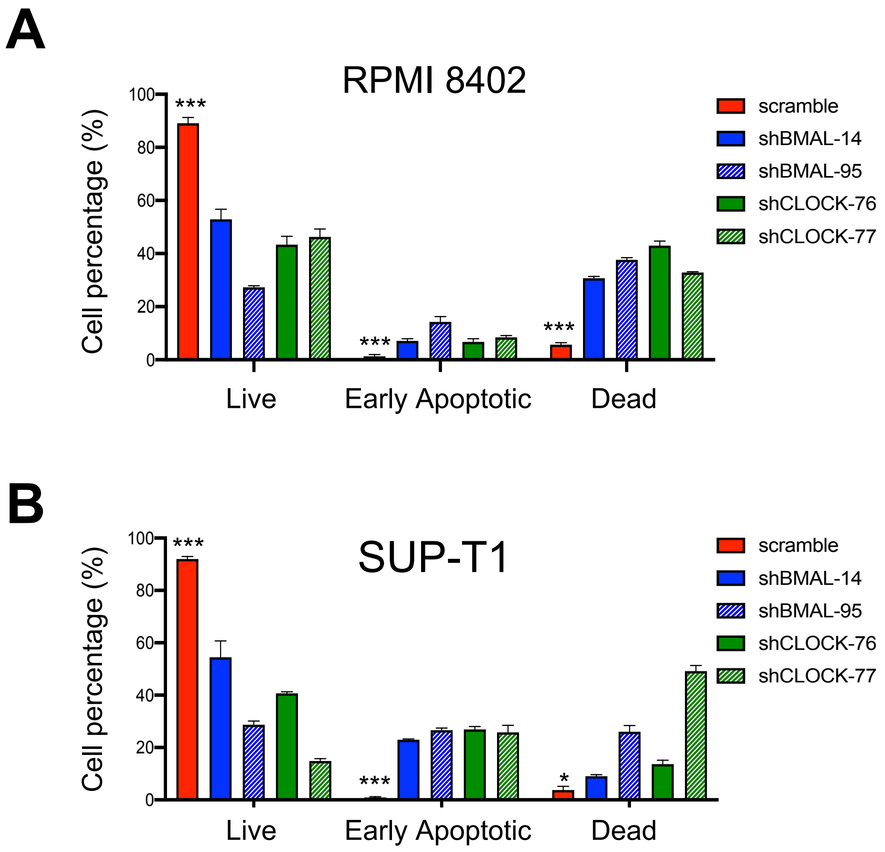

Figure S6. Apoptotic level in RPMI-8402 and SUP-T1 cells, transduced with shScramble, shBMAL1 or shCLOCK lentivectors.

(A-B) Flow cytometric analysis of apoptotic level by AnnexinV binding and 7AAD exclusion in RPMI-8402 (**A**) and SUP-T1 (**B**) cells after transduction with shBMAL1 and shCLOCK constructs or scramble control as indicated. AnnexinV+ 7AAD- cells were measured after seven days of *in vitro* growth by flow cytometry. The graphs report the result of two independent experiments performed in triplicate. Each reported statistical value is compared with scramble control. **, p<0.05; **, p<0.01; ***, p<0.001 (Two-way ANOVA with Dunnett's test, comparing the sh-scramble control mean with the other values).*

Fig. S7.

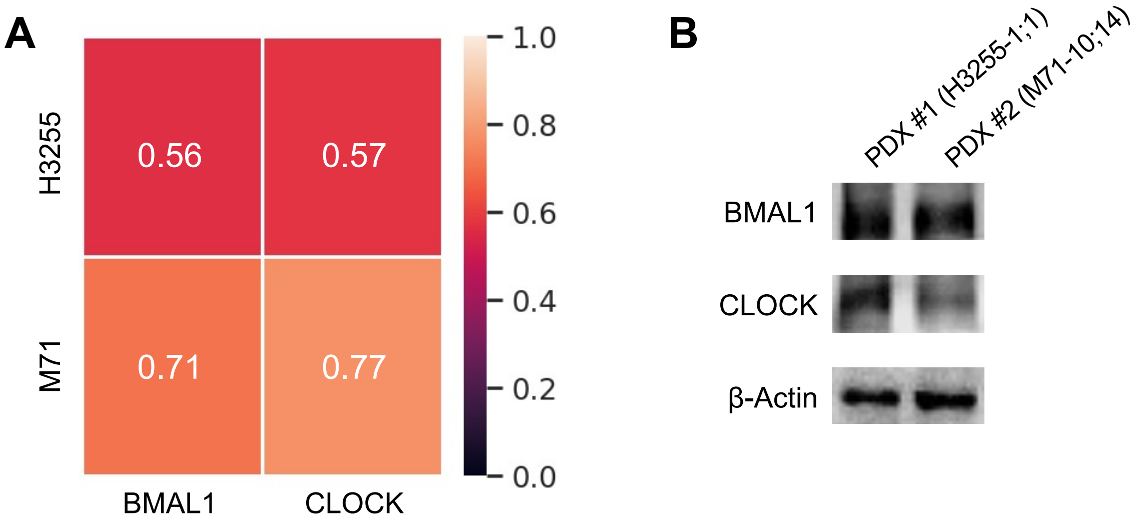

Figure S7. Expression levels of BMAL1 and CLOCK transcripts and proteins in the M71 and H3255 PDXs of T-ALL. (A) Heatmap illustrates the mRNA expression levels of ARNTL/BMAL1 and CLOCK genes in the M71 and H3255 PDXs of T-ALL. The heatmap was created using rlog transformation of RNA-seq data using a publicly available dataset (SRP103099). Normalization was done using a custom function that scales the data values to a range of 0 to 1 to better visualize the differences in expression levels. **(B)** Western blot analysis of BMAL1 and CLOCK proteins in the whole cell lysates generated from H3255-1,1 and M71-10;14 PDXs, which are derived from the *in vivo* expansion of M71 and H3255 PDXs.

Fig. S8.

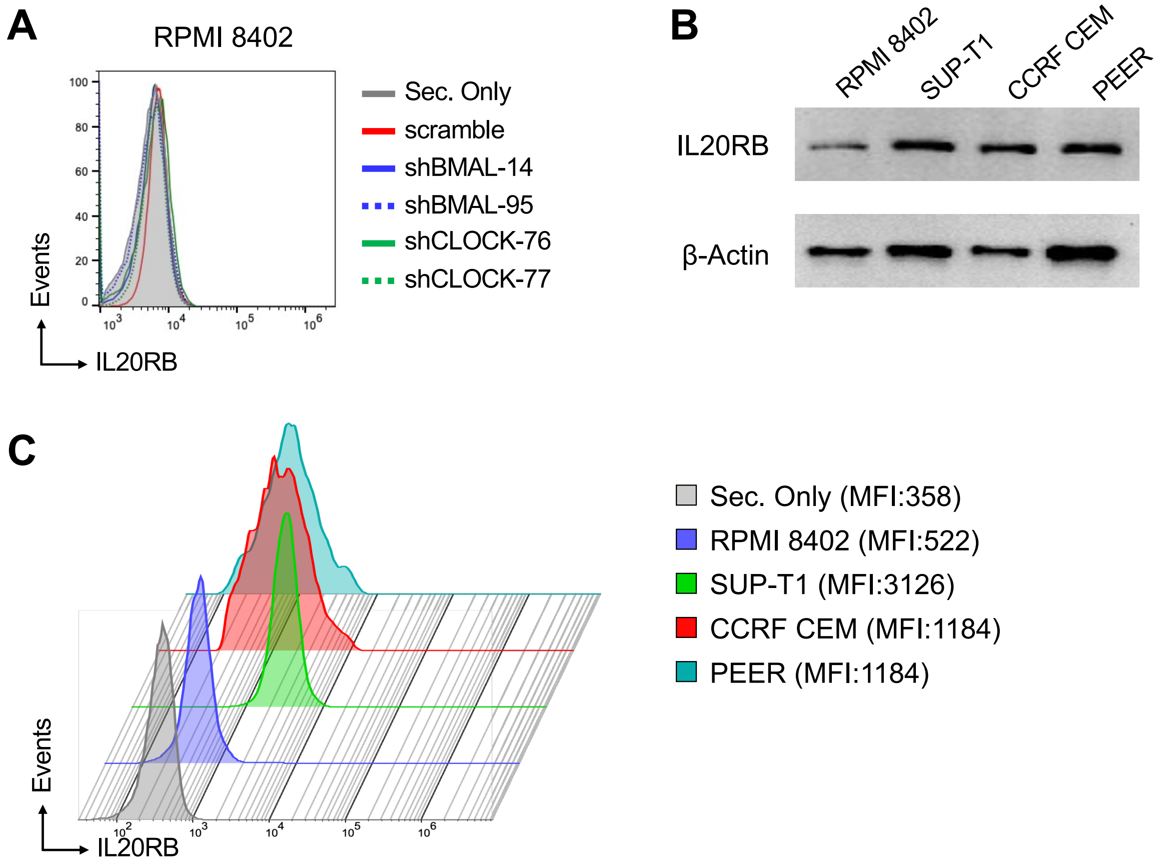

Figure S8. Protein expression level of IL20 receptor (IL20R) in RPMI-8402 T-ALL cell line by flow cytometry and western blot.

(A) Flow cytometric analysis of protein level of IL20 receptor (IL20R) in RPMI-8402 T-ALL cell line, transduced by lentiviruses encoding shRNAs against BMAL1 or CLOCK genes or scramble control. Transduced GFP+ alive cells were stained with an anti-IL20RB primary antibody and evaluated after seven days from the transduction for DAPI exclusion by flow cytometry. **(B)** Western blot analysis of IL20R protein total level in the whole cell lysates generated from RPMI-8402, SUP-T1, CCRF CEM and PEER human T-ALL cell lines. **(C)** Staggered plot showing the IL20R protein expression level in the cell surface of RPMI-8402, SUP-T1, CCRF CEM and PEER cell lines by flow cytometry. The values of mean fluorescent intensity (MFI) are reported in brackets for each sample as indicated.

Fig. S9.

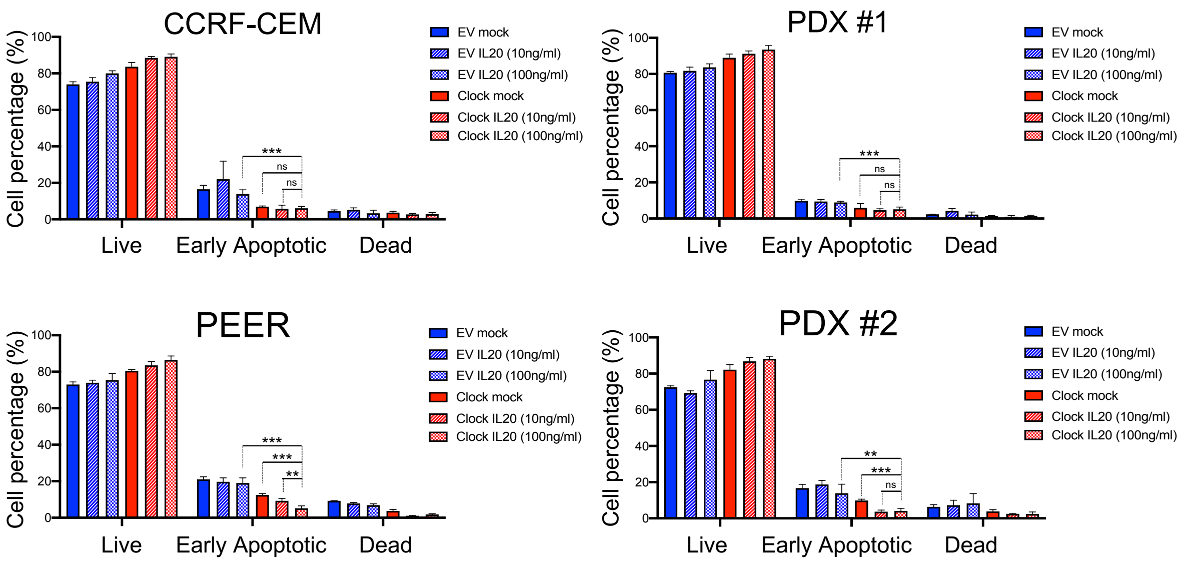

Figure S9. Apoptotic profile upon treatment of IL20 in human T-ALL cell lines and PDX samples after transduction with CLOCK or empty vector (EV) constructs. Flow cytometric analysis of apoptotic level by AnnexinV binding and 7AAD exclusion in in human CCRF-CEM and PEER cell lines and PDXs after transduction with CLOCK or empty vector (EV) constructs as indicated. AnnexinV+ 7AAD- transduced cells were measured after two days of in vitro treatments with IL20 at the indicated concentration (10 ng/ml and 100 ng/ml) or phosphate-buffered saline (PBS) as mock control by flow cytometry. The graphs report the result of two independent experiments performed in triplicate. *ns, not significant; **, $p<0.05$; **, $p<0.01$; ***, $p<0.001$ (Student's t-test).*

Fig. S10.

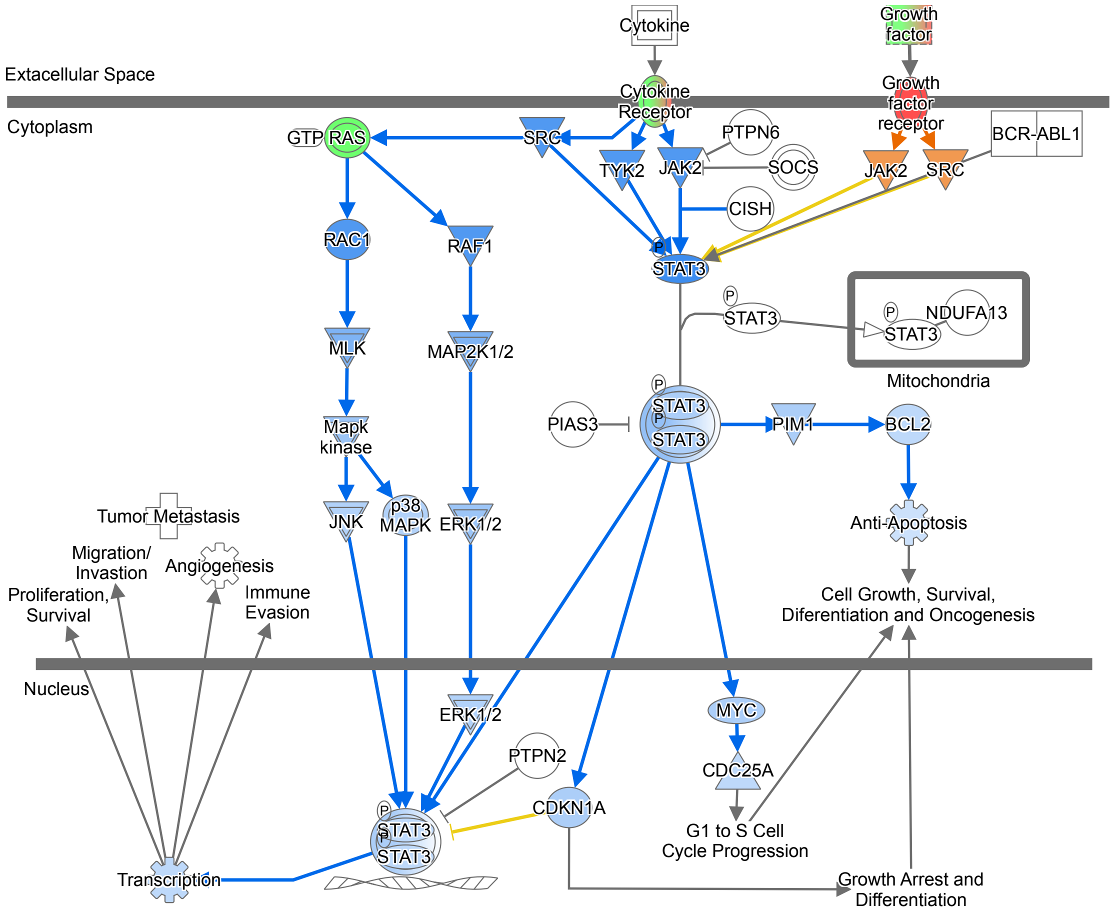

Figure S10. CLOCK and BMAL1 transcription factors promote the expression of genes involved in JAK/STAT signaling pathway in T-ALL. Pathway analysis of differentially expressed genes in shBMAL1/CLOCK vs. shScramble conditions in T-ALL cells. The network graph was obtained by IPA. In blue/green, downregulated genes. In red/orange, upregulated genes. Blue lines signify predicted consistently regulated genes while yellow lines highlight predicted inconsistent gene expression regulation.

Fig. S11.

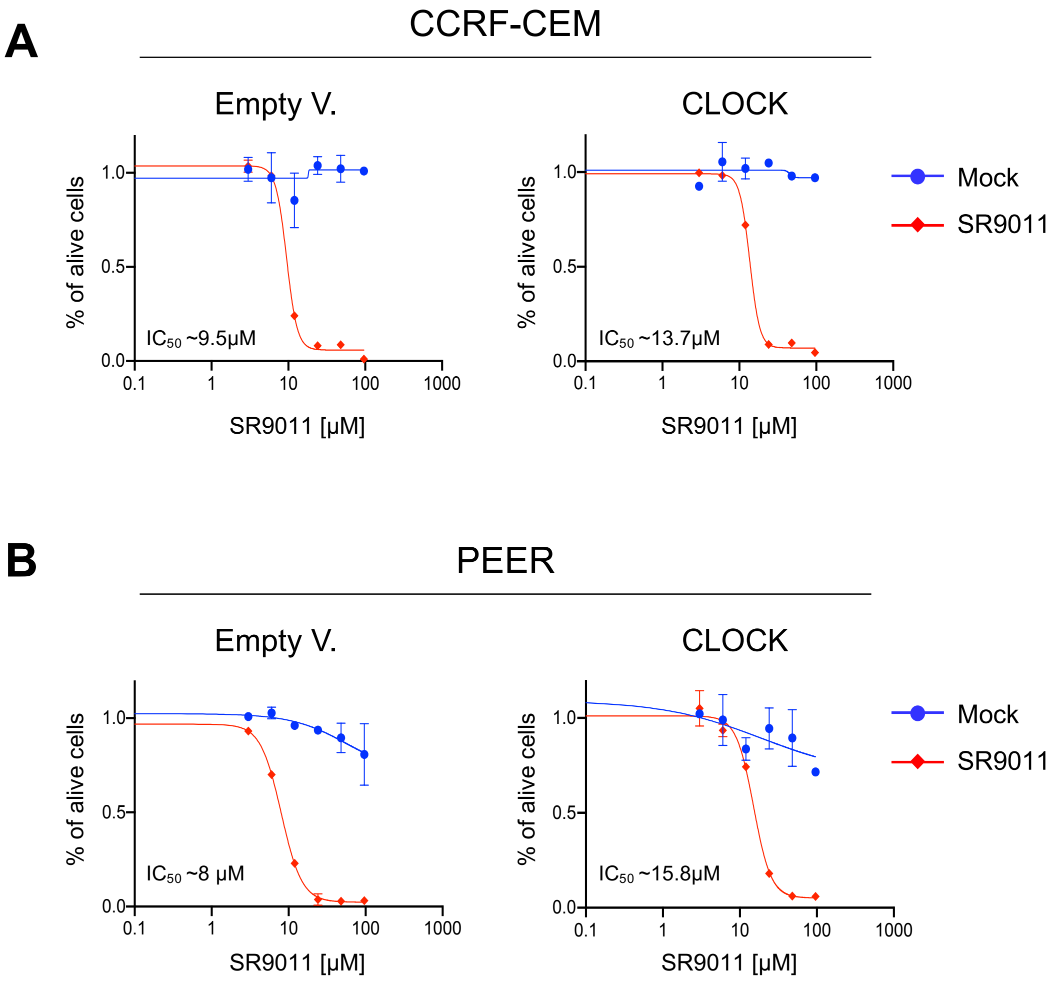

Figure S11. Representative lethal dose-response curve for SR9011, agonist of the nuclear hormone receptors Rev-Erbα and Rev-Erbβ. Cytotoxicity assays were performed on CCRF-CEM and PEER T-ALL cell lines, overexpressing CLOCK gene function or empty vector (EV) as control. Viable cells were measured after 72h of treatment with DMSO (mock) or SR9011 at the indicated concentrations by flow cytometry analysis for DRAQ7 exclusion. The graphs report the result of two independent experiments performed in duplicates (mean ± SD are plotted).

Supplementary Tables

Table S1.

Donor (clone)	Donor Type	Recipient ID (Strain)	Injected Cells after FACSort (% purity)	Injected Cell Dose	Clinical Outcome	Latency (days)	% CD45+ Spleen Cells at Necropsy	GFP+ Leukemic Spleen Cells at Necropsy
H3255-1;1	shScamble transduced PDX	1 (NSG)	CD45+ **GFP+** (93)	150,000	Leukemia	32	nd	nd
H3255-1;1	shScamble transduced PDX	2 (NSG)	CD45+ **GFP+** (93)	150,000	Leukemia	32	53	88
H3255-1;1	shScamble transduced PDX	3 (NSG)	CD45+ **GFP+** (93)	150,000	Leukemia	32	43	74
H3255-1;1	shScamble transduced PDX	4 (NSG)	CD45+ **GFP+** (93)	150,000	Leukemia	42	nd	nd
H3255-1;1	shScamble transduced PDX	5 (NSG)	CD45+ **GFP+** (93)	15,000	Leukemia	50	nd	nd
H3255-1;1	shScamble transduced PDX	6 (NSG)	CD45+ **GFP+** (93)	15,000	Leukemia	77	54	89
H3255-1;1	shScamble transduced PDX	7 (NSG)	CD45+ **GFP+** (93)	15,000	NED (d150)			
H3255-1;1	shScamble transduced PDX	8 (NSG)	CD45+ **GFP+** (93)	15,000	NED (d150)			
H3255-1;1	shCLOCK_76 transduced PDX	1 (NSG)	CD45+ **GFP+** (92)	150,000	Leukemia	67	48	59
H3255-1;1	shCLOCK_76 transduced PDX	2 (NSG)	CD45+ **GFP+** (92)	150,000	Leukemia	54	63	67
H3255-1;1	shCLOCK_76 transduced PDX	3 (NSG)	CD45+ **GFP+** (92)	150,000	NED (d150)			
H3255-1;1	shCLOCK_76 transduced PDX	4 (NSG)	CD45+ **GFP+** (92)	150,000	NED (d150)			
H3255-1;1	shCLOCK_76 transduced PDX	5 (NSG)	CD45+ **GFP+** (92)	15,000	NED (d150)			
H3255-1;1	shCLOCK_76 transduced PDX	6 (NSG)	CD45+ **GFP+** (92)	15,000	Leukemia	87	56	88
H3255-1;1	shCLOCK_76 transduced PDX	7 (NSG)	CD45+ **GFP+** (92)	15,000	NED (d150)			
H3255-1;1	shCLOCK_76 transduced PDX	8 (NSG)	CD45+ **GFP+** (92)	15,000	NED (d150)			
H3255-1;1	shCLOCK_77 transduced PDX	1 (NSG)	CD45+ **GFP+** (95)	150,000	Leukemia	43	75	77
H3255-1;1	shCLOCK_77 transduced PDX	2 (NSG)	CD45+ **GFP+** (95)	150,000	Leukemia	59	45	54
H3255-1;1	shCLOCK_77 transduced PDX	3 (NSG)	CD45+ **GFP+** (95)	150,000	NED (d150)			
H3255-1;1	shCLOCK_77 transduced PDX	4 (NSG)	CD45+ **GFP+** (95)	150,000	NED (d150)			
H3255-1;1	shCLOCK_77 transduced PDX	5 (NSG)	CD45+ **GFP+** (95)	15,000	NED (d150)			
H3255-1;1	shCLOCK_77 transduced PDX	6 (NSG)	CD45+ **GFP+** (95)	15,000	NED (d150)			
H3255-1;1	shCLOCK_77 transduced PDX	7 (NSG)	CD45+ **GFP+** (95)	15,000	NED (d150)			
H3255-1;1	shCLOCK_77 transduced PDX	8 (NSG)	CD45+ **GFP+** (95)	15,000	NED (d150)			

**Donor (clone)**	**Donor Type**	**Recipient ID (Strain)**	**Injected Cells after FACSort (% purity)**	**Injected Cell Dose**	**Clinical Outcome**	**Latency (days)**	**% CD45+ Spleen Cells at Necropsy**	**GFP+ Leukemic Spleen Cells at Necropsy**
M71-10;14	shScamble transduced PDX	1 (NSG)	CD45+ **GFP+** (90)	100,000	Leukemia	54	56	88
M71-10;14	shScamble transduced PDX	2 (NSG)	CD45+ **GFP+** (90)	100,000	Leukemia	54	nd	nd
M71-10;14	shScamble transduced PDX	3 (NSG)	CD45+ **GFP+** (90)	100,000	Leukemia	48	nd	nd
M71-10;14	shScamble transduced PDX	4 (NSG)	CD45+ **GFP+** (90)	100,000	Leukemia	40	58	87
M71-10;14	shScamble transduced PDX	5 (NSG)	CD45+ **GFP+** (90)	20,000	Leukemia	54	64	89
M71-10;14	shScamble transduced PDX	6 (NSG)	CD45+ **GFP+** (90)	20,000	Leukemia	48	54	84
M71-10;14	shScamble transduced PDX	7 (NSG)	CD45+ **GFP+** (90)	20,000	NED (d150)			
M71-10;14	shScamble transduced PDX	8 (NSG)	CD45+ **GFP+** (90)	20,000	NED (d150)			
M71-10;14	shCLOCK_76 transduced PDX	1 (NSG)	CD45+ **GFP+** (88)	100,000	Leukemia	48	55	57
M71-10;14	shCLOCK_76 transduced PDX	2 (NSG)	CD45+ **GFP+** (88)	100,000	NED (d150)			
M71-10;14	shCLOCK_76 transduced PDX	3 (NSG)	CD45+ **GFP+** (88)	100,000	NED (d150)			
M71-10;14	shCLOCK_76 transduced PDX	4 (NSG)	CD45+ **GFP+** (88)	100,000	NED (d150)			
M71-10;14	shCLOCK_76 transduced PDX	5 (NSG)	CD45+ **GFP+** (88)	20,000	NED (d150)			
M71-10;14	shCLOCK_76 transduced PDX	6 (NSG)	CD45+ **GFP+** (88)	20,000	NED (d150)			
M71-10;14	shCLOCK_76 transduced PDX	7 (NSG)	CD45+ **GFP+** (88)	20,000	NED (d150)			
M71-10;14	shCLOCK_76 transduced PDX	8 (NSG)	CD45+ **GFP+** (88)	20,000	NED (d150)			
M71-10;14	shCLOCK_77 transduced PDX	1 (NSG)	CD45+ **GFP+** (85)	100,000	Leukemia	64	58	65
M71-10;14	shCLOCK_77 transduced PDX	2 (NSG)	CD45+ **GFP+** (85)	100,000	NED (d150)			
M71-10;14	shCLOCK_77 transduced PDX	3 (NSG)	CD45+ **GFP+** (85)	100,000	NED (d150)			
M71-10;14	shCLOCK_77 transduced PDX	4 (NSG)	CD45+ **GFP+** (85)	100,000	NED (d150)			
M71-10;14	shCLOCK_77 transduced PDX	5 (NSG)	CD45+ **GFP+** (85)	20,000	NED (d150)			
M71-10;14	shCLOCK_77 transduced PDX	6 (NSG)	CD45+ **GFP+** (85)	20,000	NED (d150)			
M71-10;14	shCLOCK_77 transduced PDX	7 (NSG)	CD45+ **GFP+** (85)	20,000	NED (d150)			
M71-10;14	shCLOCK_77 transduced PDX	8 (NSG)	CD45+ **GFP+** (85)	20,000	NED (d150)			

Table S1, related to Figure 2. Summary of transplant experiments with H3255-1;1 (PDX #1) and M71-10;14 (PDX #2), after transduction with shCLOCK or shScramble co-expressed with GFP from single lentiviral vectors.

% CD45+ cell fraction is among gated viable spleen cells; % GFP+ is among gated viable human CD45+ cells.

NED (dX), no evidence of disease as of X days post-transplant; ND, not determined.

Table S2, related to Figure 3. Top gene transcripts (N=622) differentially expressed upon shBMAL1 in RPMI-8402.

Table S3, related to Figure 3. Top gene transcripts (N=721) differentially expressed upon shCLOCK in RPMI-8402

Table S4, related to Figure 3. Top gene transcripts, highly down-regulated in the shCLOCK/BMAL1-transduced cells as compared to control cell conditions and bound by BMAL1 in RPMI-8402 cell line.
